# Long lasting control of viral rebound with a new drug ABX464 targeting Rev – mediated viral RNA biogenesis

**DOI:** 10.1186/s12977-015-0159-3

**Published:** 2015-04-09

**Authors:** Noëlie Campos, Renier Myburgh, Aude Garcel, Audrey Vautrin, Laure Lapasset, Erika Schläpfer Nadal, Florence Mahuteau-Betzer, Romain Najman, Pauline Fornarelli, Katjana Tantale, Eugénia Basyuk, Martial Séveno, Julian P Venables, Bernard Pau, Edouard Bertrand, Mark A Wainberg, Roberto F Speck, Didier Scherrer, Jamal Tazi

**Affiliations:** ABIVAX, 1919 route de Mende, 34293 Montpellier Cedex 5, France; Institut de Génétique Moléculaire de Montpellier, University of Montpellier, CNRS UMR 5535, 1919 route de Mende, 34293 Montpellier Cedex 5, France; Division of Infectious Diseases and Hospital Epidemiology Department of Internal Medicin, University of Zurich, University Hospital, Raemistrasse 100, 8091 Zurich, Switzerland; Institut Curie, CNRS UMR9187, INSERM U1196, Centre universitaire, Bâtiment 110, 15 rue Georges Clémenceau, 91405 ORSAY CEDEX, France; Université de Montpellier, UFR Pharmacie, 15 Avenue Charles Flahault, 34000 Montpellier, France; McGill AIDS Center, Lady Davis Institute – Jewish General Hospital, Montréal, QC Canada; Plate-forme de Protéomique Fonctionnelle (FPP) IGF, UMR 5203 CNRS - INSERM U661- UM, 141 rue de la Cardonille (pièce 029), 34094 Montpellier CEDEX 05, France

**Keywords:** AIDS, RNA biogenesis, Splicing, New antiviral drug, HIV cure, Cap Binding Complex, Rev protein

## Abstract

**Background:**

Current therapies have succeeded in controlling AIDS pandemic. However, there is a continuing need for new drugs, in particular those acting through new and as yet unexplored mechanisms of action to achieve HIV infection cure. We took advantage of the unique feature of proviral genome to require both activation and inhibition of splicing of viral transcripts to develop molecules capable of achieving long lasting effect on viral replication in humanized mouse models through inhibition of Rev-mediated viral RNA biogenesis.

**Results:**

Current HIV therapies reduce viral load during treatment but titers rebound after treatment is discontinued. We devised a new drug that has a long lasting effect after viral load reduction. We demonstrate here that ABX464 compromises HIV replication of clinical isolates of different subtypes without selecting for drug resistance in PBMCs or macrophages. ABX464 alone, also efficiently compromised viral proliferation in two humanized mouse models infected with HIV that require a combination of 3TC, Raltegravir and Tenofovir (HAART) to achieve viral inhibition in current protocols. Crucially, while viral load increased dramatically just one week after stopping HAART treatment, only slight rebound was observed following treatment cessation with ABX464 and the magnitude of the rebound was maintained below to that of HAART for two months after stopping the treatment. Using a system to visualize single HIV RNA molecules in living cells, we show that ABX464 inhibits viral replication by preventing Rev-mediated export of unspliced HIV-1 transcripts to the cytoplasm and by interacting with the Cap Binding Complex (CBC). Deep sequencing of viral RNA from treated cells established that retained viral RNA is massively spliced but importantly, normal cellular splicing is unaffected by the drug. Consistently ABX464 is non-toxic in humans and therefore represents a promising complement to current HIV therapies.

**Conclusions:**

ABX464 represents a novel class of anti-HIV molecules with unique properties. ABX464 has a long lasting effect in humanized mice and neutralizes the expression of HIV-1 proviral genome of infected immune cells including reservoirs and it is therefore a promising drug toward a functional cure of HIV.

**Electronic supplementary material:**

The online version of this article (doi:10.1186/s12977-015-0159-3) contains supplementary material, which is available to authorized users.

## Background

AIDS is a worldwide pandemic. Current therapies have succeeded in controlling the disease but long-term use of Anti-Retroviral Therapy (ART), is limited by issues of drug resistance and side effects [1-3]. Furthermore, the current ART drugs need to be taken for life time and only attenuate the disease without curing it [4]. Therefore, there is a continuous need for new drugs to HIV cure, in particular those acting through new and as yet unexplored mechanisms of action [5].

We took advantage of the unique feature of the viral genome once integrated in infected cells to require both activation and inhibition of splicing of precursor mRNAs to develop molecules capable of changing the balance between spliced and unspliced products. The HIV-1 DNA genome expresses a primary transcript of 9 kilobases (kb) that not only serves as genomic RNA for progeny virus but also as the mRNA that encodes the viral Gag and Gag-Pol proteins [6-8]. Alternative splicing is a key event for HIV replication. Successful infection and production of new infectious viruses requires the balanced expression of seven additional viral proteins (Rev, Tat, Nef, Vif, Vpr, Vpu and Env) that are produced by splicing of the primary 9 kb transcripts among which the Tat and the Rev factors are absolutely required for viral gene expression at the transcriptional and post-transcriptional levels in infected cells [9,10]. While most cellular unspliced RNAs are retained in the nucleus where they are degraded, nuclear export of the unspliced viral RNAs is facilitated by the Rev protein, through binding to a viral sequence called the Rev responsive element (RRE) [9,11,12].

After screening a collection of chemical compounds, one indole derivative (IDC16) was discovered to interfere with splicing enhancer activity of the SR protein splicing factor SRSF1 [8,13,14]. This compound suppresses the production of key viral proteins, thereby compromising subsequent synthesis of full-length HIV-1 pre-mRNA and assembly of infectious particles. However, IDC16 is a planar fused tetracyclic indole compound. Such molecules, and in particular those in which the polyaromatic nucleus is further substituted by a positively charged (protonated aminoalkyl) side chain, have been studied as potential anticancer agents [15,16]. The guiding principle is that they intercalate DNA and exhibit cytotoxic effects by interfering with the function of DNA processing enzymes such as topoisomerase I and II [17,18]. We thus designed alternative scaffolds which retain the structural characteristics of IDC16 but which lose the inherent affinity of the flat polycyclic molecules for DNA. We prepared molecules that are more flexible as they have fewer fused rings. This could circumvent potential side effects and allow a more optimal interaction with the protein target. A dedicated library of 1,000 compounds of potentially more potent and selective splicing modulators was synthesized.

Here, we describe ABX464, an enhancer of viral RNA splicing with validated efficacy in humanized mouse models of HIV-1 infection. ABX464 is the first therapeutic molecule described to neutralize the expression of the HIV-1 proviral genome and it is therefore a promising drug towards HIV cure.

## Results

### Selection of ABX464

**Figure 1:**
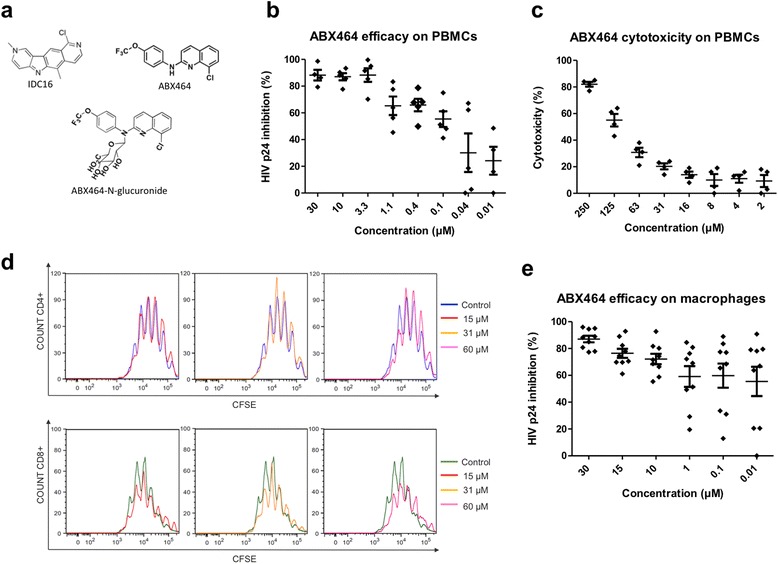
**ABX464 Inhibits HIV-1 production in PBMC- and macrophages-infected cells. a** Drawing of 10-chloro-2,6-dimethyl-2H-pyrido[3′,4′:4,5]pyrrolo[2,3-g]isoquinoline (IDC16), 8-chloro-*N-*(4-(trifluoromethoxy)phenyl)quinolin-2-amine (ABX464) and 8-chloro-*N-*glucuronide-*N*-(4-(trifluoromethoxy)phenyl)quinolin-2-amine) (ABX464-*N*-glucuronide) compounds. **b** HIV-1 strain Ada-MR5 was used to infect triplicate of activated PBMCs from different donors (stimulated for two days with PHA and IL2) in the absence or presence of increasing concentrations of ABX464. Supernatant was harvested 6 days post-infection (pi) and viral capsid protein p24 antigen was quantitated using a standard ELISA protocol. Each point represents 5 donors. **c** Concurrently, cell viability was measured by MTS assay after 6 days of incubation and cytotoxicity was indicated as percentage as compared with untreated cells. **d** Histogram plots of CFSE fluorescence of CD4^+^ (upper panel) or CD8+ (lower panel) after 3 days culture without (blue and green curves, respectively) or with increasing concentrations of ABX464; 15 μM (red curves), 31 μM (yellow curves) or 61 μM (pink curves). CD4^+^ and CD8+ T cells purified from PBMCs were labeled with CFSE and cultured with the indicated concentration of ABX464 and then analysed by flow cytometry. The plots show the CFSE profiles of viable CFSE-labeled CD4^+^ and CD8+ T cells. **e** HIV-1 strain YU2 was used to infect triplicate of monocyte-derived macrophages from different donors in the absence or presence of increasing concentrations of ABX464. Supernatant was harvested 8 days pi and viral capsid protein p24 antigen was quantitated using standard ELISA protocol. Each point represents 9 donors.

The first functional screening of the new compounds was based on the use of freshly isolated human peripheral blood mononuclear cells (PBMCs) from healthy donors. These PBMCs were infected by the laboratory HIV strain Ada-MR5. Every molecule was tested at 5 μM on at least 8 different blood isolates; for a rigorous selection of candidate molecules, we maintained the cells with or without the drug for 6 days. Five compounds were selected following these assays and they were demonstrated to have an IC50 in the micromolar range. We selected one, ABX464 (Figure 1a) for further analysis. However, ABX464 is hydrophobic and forms aggregates when applied directly to cell cultures, which leads to some toxicity to the cells (data not shown). To avoid this problem, we prepared a soluble fraction after adding the molecule to cell culture media and elimination of aggregates by centrifugation (see Methods). The binding of ABX464 to bovine serum albumin (BSA) retains the molecule in the soluble fraction as determined by mass spectrometry. Figure 1b, shows dose dependent inhibition of HIV-1 replication by soluble ABX464 in stimulated PBMCs from 5 different donors with an IC50 ranging between 0.1 μM and 0.5 μM. We also determined the concentration of ABX464 with minimal side effects on cell viability using an MTS test (Figure 1c) and cell proliferation was measured by the stable incorporation of the intracellular fluorescent dye 5-(and −6)-carboxyfluorescein diacetate succinimidyl ester (CFSE) into lymphocytes (Figure 1d). ABX464 is only toxic at doses higher than 60 μM but has no effect on cell proliferation.

To generalize the effect of ABX464 on HIV-1 replication in other primary cells, the same protocol was repeated using infected macrophages, which act as viral reservoirs. Cells were treated with between 0.01 μM up to 30 μM concentrations of ABX464 and p24 antigen levels were monitored in culture supernatants over a 12 days period (Figure 1e). Interestingly, ABX464 efficiently blocked virus replication in a dose-dependent manner with an IC50 ranging between 0.1 μM and 1 μM. However, cell viability was not decreased under ABX464 treatment (data not shown).

### ABX464 does not select for HIV specific mutations and it is not genotoxic

**Figure 2:**
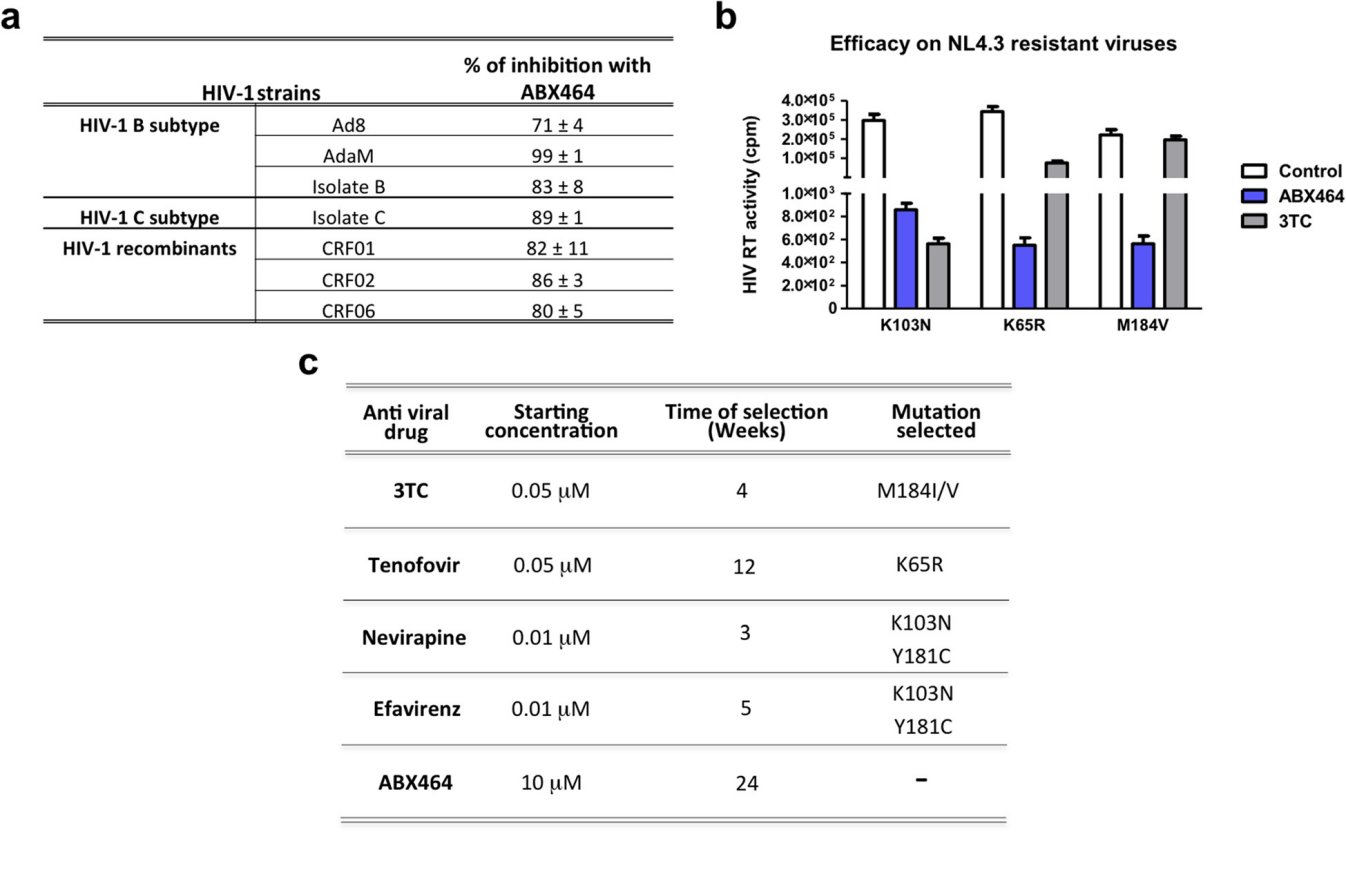
**ABX464 inhibits different HIV-1 clades, resistant viruses and did not select for resistance. a** Different HIV-1 strains (clade B, clade C and recombinants clades) were used to infect PBMCs from three different donors in the absence or presence of 5 μM of ABX464. Supernatant was harvested 6 days pi and viral capsid protein p24 antigen was quantitated using standard ELISA protocol. **b** RT activity (cpm) measured in human PBMCs infected with different resistant mutants of NL4.3 strain (K103N, K65R and M184V) and treated with ABX464 or 3TC. **c** Resistance to ABX464 was tested on human PBMCs and compared to current therapies (see Methods). There were no resistance-inducing mutations detected after treatment with ABX464 for at least 24 weeks.

To complement the previous experiments, which were all performed with primary human cells infected with macrophage-tropic (R5) strains (Ada-MR5 and YU2), we shifted to an *in vitro* system that may be more relevant to the clinical situation. By infecting primary cells with HIV-1 isolates from patients, we showed that ABX464 had a strong inhibitory effect for all HIV-1 subtypes tested including subtype B, C and recombinant viruses (Figure 2a).

A critical aspect of HIV infection is its ability to generate a diverse viral population through high replication rates and high reverse transcriptase mutation rates. Thus, in infected individual there is a broad pool of virus that may survive in the face of pressure exerted from the host as well as non-potent antiretroviral therapy. This same mechanism is responsible for the selection of drug resistance. ABX464 also very efficiently inhibited the replication of viral strains harbouring mutations that confer resistance to different therapeutic agents *in vitro* (Figure 2b). While the antiviral drug 3TC was not highly active on K65R and M184V mutant strains, both strains were inhibited by ABX464.

Genetic heterogeneity is a characteristic of HIV, which contributes significantly to its ability to generate mutations that overcome the efficacy of drug therapies. The selection of drug resistant mutants *in vitro* can be readily accomplished by maintaining the virus in a state of sub-optimal growth, regulated by slowly increasing the amount of drug pressure applied. Resistance to ABX464 was tested on human PBMCs and compared to current therapies (Figure 2c). There were no resistance-inducing mutations detected after treatment with ABX464 for at least 24 weeks (Figure 2c).

We also applied a deep sequencing approach for sensitive detection of low-frequency viral variants across the entire HIV-1 genome. Viruses derived from treated and untreated infected primary macrophages of 4 different donors were sequenced and reads not aligning to human genome were aligned to YU2 sequence using GSNAP [19] (Raw data are provided upon request). The majority of low and high frequency mutations were equally present in treated and untreated samples, demonstrating that ABX464 does not select for specific mutations (Additional file 1: Figure S1a). To ascertain that amplification of viruses from treated samples will not mutate when amplified in PBMCs, they were sequenced following amplification with or without drug pressure. Again, no novel mutations were detected other than the ones existing before treatment in the original samples (Additional file 1: Figure S1b). We conclude that ABX464 was unlikely to select for specific viral mutations that might inhibit viral replication.

The potential genotoxicity of ABX464 was also assessed in GLP-compliant *in vitro* and *in vivo* studies and in neither case was ABX464 is genotoxic (see below, Additional file 2: Table S1). This is making clear improvement to fulfill criteria for clinical development, unlike IDC16.

### ABX464 increases the levels of spliced HIV RNA

Since the parent drug IDC16 has a specific action on HIV-1 pre-mRNA splicing, we assessed the efficiency of ABX464 using the p∆PSP plasmid. p∆PSP containing the HIV-1 proviral genome which is deleted between nucleotides 1,511 and 4,550 (Additional file 3: Figure S2a) and this plasmid recapitulates all splicing events of HIV-1 pre-mRNA in transfected HeLa cells [13]. The mRNAs produced by splicing were then analysed by RT-PCR using forward and reverse primers that amplify several differentially spliced isoforms encoding the viral proteins Nef, Rev, and Tat. To assess whether ABX464 induced preferential selection of a few splice sites and will favour the production of a specific viral RNA, amplified products were visualized and analysed on the LabChip HT DNA assay on an automated microfluidic station (Caliper, Hopkinton, MA). Each band corresponding to specific viral RNA was compared to total RNA amplified using Caliper software. Unlike IDC16 which was previously shown to completely block the production of spliced viral RNA isoforms [13], ABX464 did not alter the splicing profile when used at concentrations of 5 μM or 10 μM (Additional file 3: Figure S2b). Besides, there was no significant variation in the levels of each specific splice variants between treated and untreated samples (Additional file 3: Figure S2c).

**Figure 3:**
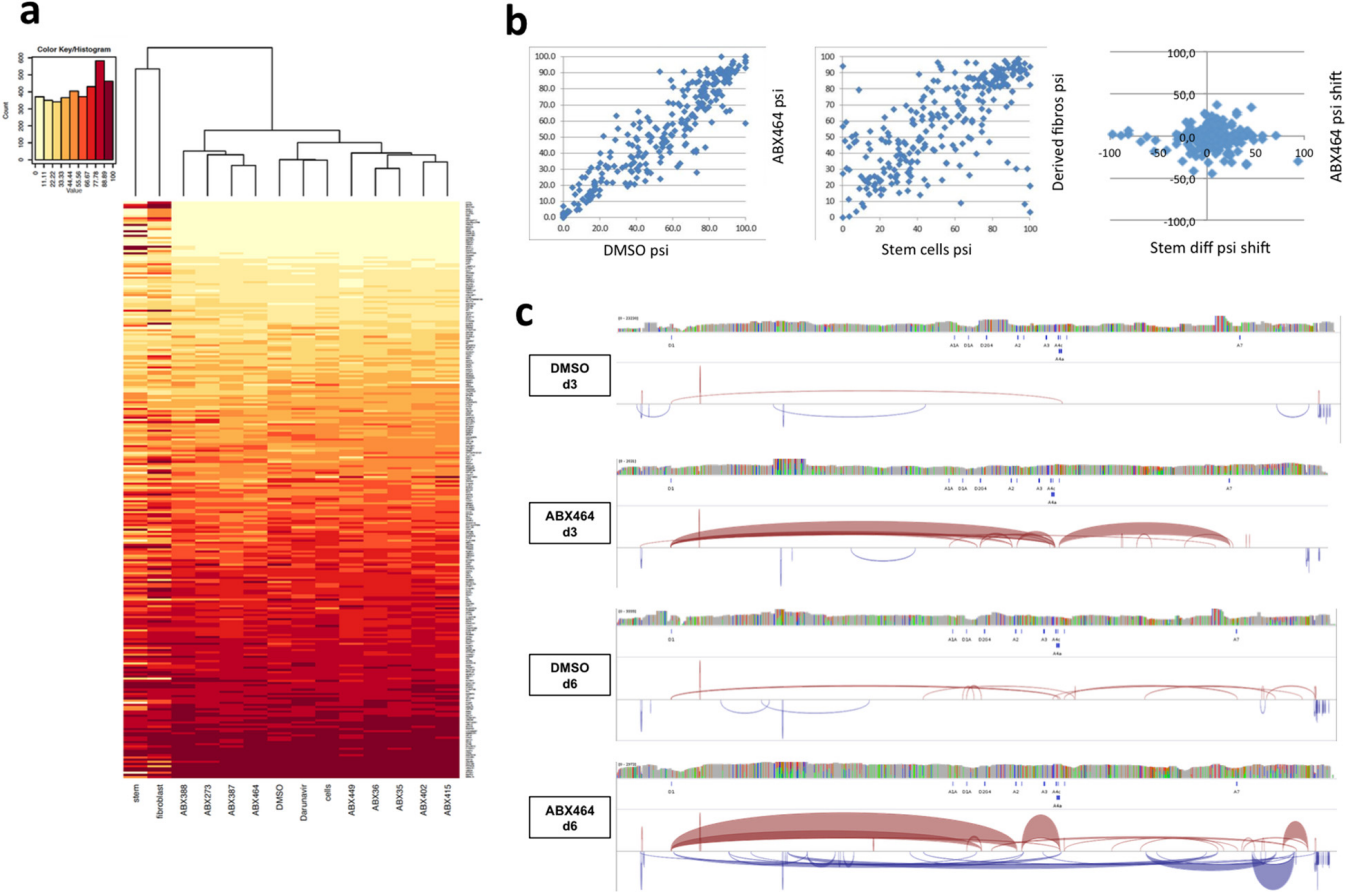
**Effect of ABX464 on cellular and HIV-1 RNA splicing. a** Heat map showing percent-spliced-in (psi) shifts for the 264 alternate splicing events in positive control fibroblasts (fibroblasts) and their iPSCs (stem) untreated, PBMCs untreated (Cells) or PBMCs treated by either DMSO without drug (DMSO) or with various compounds (ABX35, ABX36, ABX273, ABX388, ABX387 ABX402, ABX415, ABX449, ABX464 and Darunavir). **b** Scatter plots comparing splicing changes for the 264 exons in PBMCs treated with ABX464 versus DMSO (left panel) or stem cells differentiated into fibroblast (middle panel). The right panel shows a scatter plot comparing psi changes for the 264 exons induced by ABX464 to those induced during stem cell differentiation. Pearson correlations (R values) are shown. **c** Visualisation of HIV-1 splice junctions after capture and sequencing of HIV-1 RNAs extracted from PBMCs infected with the YU2 strain, either untreated (DMSO) or treated with ABX464 (ABX464) 3 days pi (d3) or 6 days pi (d6) using 454 pyrosequencing (according to GS junior method manual). Estimate of the distribution of read mappings, positions of known acceptor (A1, A2, A3, A4a, A4c and A7) and donor splice sites (D1a, D2G4, D3 and D4), the exon-exon junctions (the line of accolade which is dependent of the junction abundance) and the known coding regions of HIV-1, are shown.

In order to verify that ABX464 did not significantly or globally affect the splicing events of endogenous genes, which could potentially lead to some adverse consequences, the effect of ABX464 was tested by RT-PCR analysis on a pre-existing panel of 382 alternative splicing events (ASEs). These 382 ASEs represent a high-throughput (HT) random snapshot of global alterations of alternative splicing. We performed HT-PCR analysis of these (essentially random) 382 ASEs on multiple PBMC samples, with nine of our drug derivatives, including ABX464, and with various controls: either untreated or treated with DMSO or the control antiviral drug (Darunavir) [20,21]. Analysis of the data allowed further stringent quality controls; ASEs were only considered if >75% of the products ran at the expected mobilities and if total expected PCR concentration was higher than 20 nM. This filter ensured the use of high quality PCRs on well-expressed genes and led to 264 remaining ASEs in our analysis. The splicing profiles of the 12 PBMC samples are shown in Figure 3a compared with stem cells and their derived fibroblasts (a previous treatment that had previously been shown to result in widespread splicing changes) [22]. This shows that there was very little difference between the splicing profiles of the drug-treated PBMC samples and controls, as they formed one of three separate poles with the stem cells and their derived fibroblasts. Consistent with this, the untreated cells and ABX464 treated cells per cent spliced in values for these 264 ASEs had a high correlation of R = 0.89, whereas stem cells and derived fibroblasts correlated poorly at R = 0.59 (Figure 3b). Taken together these data show that ABX464 had a minimal or no global effect on pre-mRNA splicing.

To test whether ABX464 influenced the splicing of HIV RNA in infected cells, we performed an array-based sequence capture using a customized library probes targeting HIV sequences to get rid of cellular RNA. The probes were used to capture cDNAs prepared from infected treated and untreated PBMCs. After double capture, libraries were prepared and sequenced using 454 pyrosequencing (according to GS junior method manual). The average size of the reads around 400 bp allowed unambiguous assembly of viral genome from untreated sample (after 3 and 6 days of infection) using reads that were not mapped to human genome (hg19) (Figure 3c). All sequencing data were analysed using GSNAP [19]. After 3 days post-infection we obtained 32,289 reads for the untreated DMSO sample and 4149 reads for ABX464 treated sample. Strikingly, 17.4% of the reads from treated sample corresponded to splice junctions, against 0.93% in the untreated sample. While the number of reads from treated and untreated samples were similar at 6 days post-infection (20,585 and 27,984, respectively), the fraction corresponding to splice junctions was again larger in treated (13.3%) compared to untreated sample (1.93%). Based on these results we conclude that ABX464 favoured spliced HIV RNA in infected PBMCs, which compromised subsequent synthesis of full-length HIV-1 pre-mRNA and assembly of infectious particles (Figure 3c). Consistently, standard procedures to measure unspliced and mutispliced RNA by quantitative RT-qPCR, demonstrated that ABX464 treatment produced 1.5 times more multispliced RNA than unspliced RNA compared to untreated infected cells (Additional file 3: Figure S2d).

In order to provide further evidence supporting the hypothesis that the anti-HIV activity we observed with ABX464 was the consequence of its inhibitory effect on viral RNA splicing after proviral DNA integration, we examined the effect of the drug on single round NL4.3R E LUC virus containing the entire HIV-1 genome mutated in the envelope gene and harbouring a luciferase marker gene in the Nef position (Additional file 3: Figure S2e). The amount of luciferase activity in cells infected with these virions reflects both the number of integrated proviruses and expression of multiply spliced species encoding Nef/Luc. As expected luciferase activity was strongly compromised with AZT treatment, as this drug blocks the synthesis of proviral DNA. In contrast, ABX464 treatment increased luciferase activity by more than 2 fold compared to untreated infected cells. These results confirmed that ABX464 acted after integration and favours the production of multispliced RNA encoding the luciferase protein.

### ABX464 interacts with CBC complex and prevents Rev-mediated export of unspliced viral RNA

**Figure 4:**
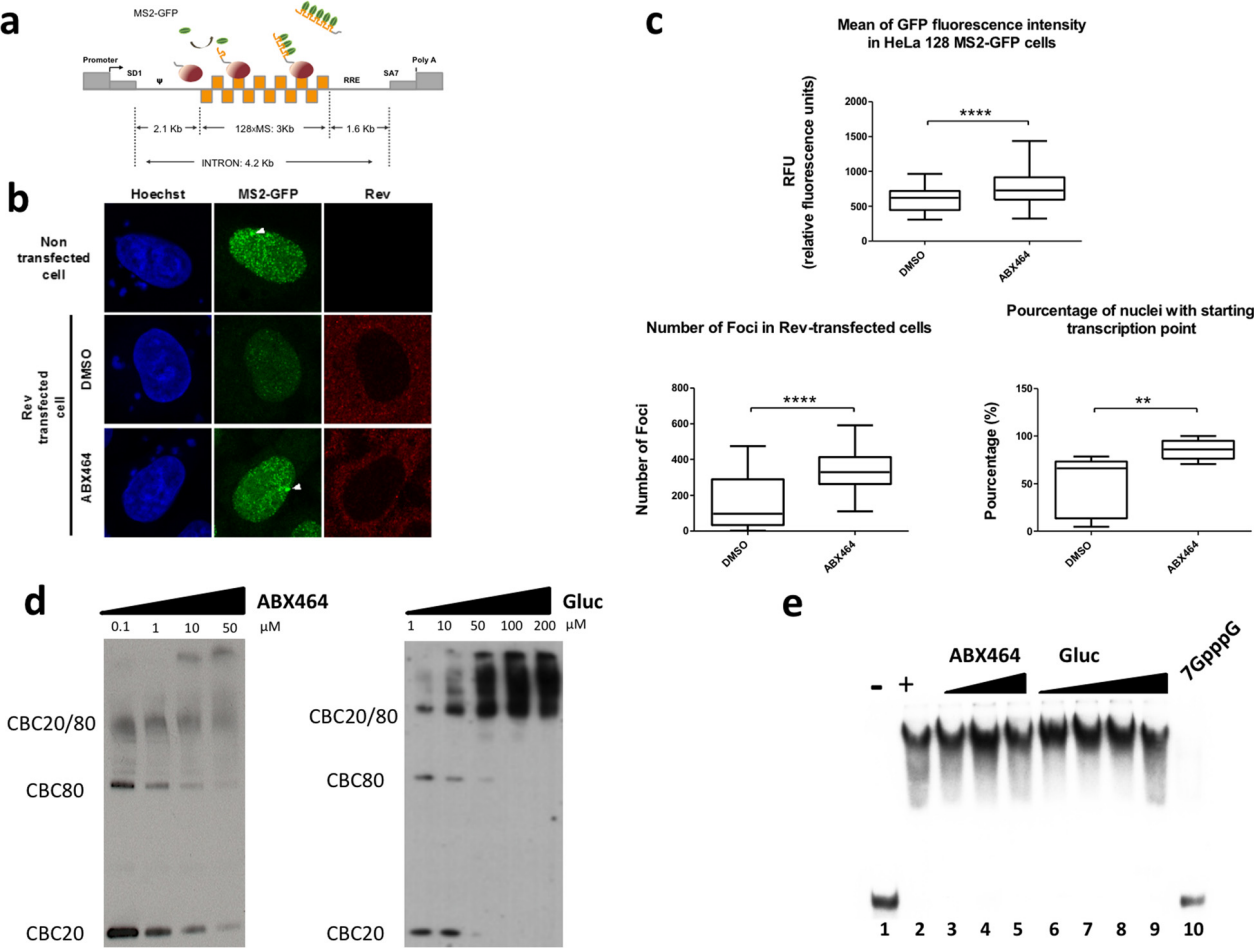
**ABX464, influences REV-mediated HIV RNA biogenesis and export, and interacts with the CBC complex. a** Schematic representation of the HIV-1 reporter gene. **b** Visualization of GFP-MS2-Bound RNA in HeLa cells expressing Tat, MS2-GFP and HIV-reporter transcripts in non-transfected or transfected cells with Rev. Rev-transfected cells were untreated (DMSO) or treated with ABX464 (ABX464). Arrow indicates transcription site. **c** Quantification of images corresponding to untreated (DMSO) or treated (ABX464) in (b) of total GFP (upper panel), in the nucleoplasm (left panel) or at the start site (right panel). Box-plots show the average GFP intensity (foci numbers or starting transcription point numbers) of Rev-transfected HeLa cells for each condition. Whiskers correspond to the minimum and maximum, boxes, to the 25–75 percentiles and the band inside the box, to the median. Statistical analysis was performed on at least 15 nuclei of Rev positive cells using an unpaired /t/-test (**: p < 0.005 and ****: p < 0.0001). **d** Purified recombinant CBC20 and CBC80 proteins were incubated with increasing concentrations of ABX464 (left panel) or ABX464-*N-*glucuronide (right panel, Gluc) and treated for 30 minutes with UV light. The proteins were revealed by Western Blotting using CBC20 and CBC80 antibodies. **e** Unlike m7GpppG cap structure, neither ABX464 nor ABX464-*N-*glucuronide interferes with the binding of capped RNA to CBC complex. Recombinant human CBC was incubated with a capped RNA substrate and analysed by native gel electrophoresis in order to resolve the different RNA and RNA-protein complexes: free RNA (lane 1), and CBC-RNA complexes (lanes 2–10) in the presence of 12 mM of m7GppG (lanes 10) or 5 μM, 10 μM or 50 μM of ABX464 (lanes 2–4, respectively) or 5 μM, 10 μM, 50 μM or 100 μM of ABX464-*N-*glucuronide (lanes 5–9, respectively).

To test the effect of ABX464 on splicing and/or export of viral RNA, we used a state of the art system to visualize single HIV RNA molecules in living cells. It is based on an HIV reporter system containing the 5′ and 3′ LTRs that harbour the promoter and polyA sites, respectively, packaging sequences and RRE elements. In addition to this, the construct contains 128 MS2 binding sites inserted between the major donor HIV-1 site (SD1) and the last splice acceptor (SA7) (Figure 4a). The reporter was introduced in HeLa cells stably expressing Tat and MS2-GFP, using the Flp-In system to create cells carrying a single copy of the transgene. Stable expression of MS2-GFP protein allowed excellent visualisation of the transcription site and single pre-mRNA molecules (Figure 4b) and did not alter splicing rate (data not shown). To assay for RNA export, we transfected these cells with constructs expressing Rev protein that will bind to the RRE and facilitate the export of unspliced viral RNA, while protecting it from the splicing machinery [23,24]. Rev expression led to a reduced GFP signal at both the transcription site and in the nucleoplasm (Figure 4b). This was expected since following association of Rev with the high-affinity RRE “nucleation site”, additional Rev molecules can polymerize along the length of the RRE in a step-wise fashion through both protein-protein and protein-RNA interactions [25], thereby removing MS2-GFP from their target sequences. Rev-mediated RNA export will also lead to a reduction of unspliced RNA in the nucleus and a reduction in the intensity of GFP in the nucleus. More nuclear GFP signal was observed in Rev transfected cells treated with ABX464 compared to untreated cells (Figure 4 b and c upper panel). Crucially, ABX464 interfered with both activities of Rev by preserving the GFP signal both at the transcription site (Figure 4 b and c lower left panel) and in the nucleoplasm of cells expressing Rev (Figure 4 b and c lower right panel). However, ABX464 showed no effect on reporter cells in the absence of Rev (Additional file 4: Figure S3).

Both, RNA export and RNA splicing are controlled by the cap binding complex (CBC) which interacts directly with either Rev or the transcription/export (TREX) complex, a multi-protein complex, required for transcription and export of bulk mRNAs [26]. These interactions are thought to recruit Rev and TREX to a region near the 5′-terminal cap structure of mRNA [27] and thereby connect the transcription and export of newly transcribed RNAs. Since ABX464 interfered with Rev-mediated functions, it was important to test whether ABX464 bounds to either Rev or the CBC complex. Using a derivative of ABX464 that has a photoactivatable moiety and competition with ABX464 on purified recombinant CBC20 and CBC80 (CBC) [28], we discovered that ABX464, itself, was able to induce dose-dependent covalent bridging between CBC20 and CBC80, after UV irradiation and this complex could be resolved by SDS-PAGE (Figure 4d and Additional file 5: Figure S4). Mass spectrometry analysis of gel-purified CBC20, CBC80 and the putative complex CBC (80 and 20), showed that the trypsin digestion of CBC (80 and 20) complex gave rise to all predicted peptides except the peptide corresponding to the position 37–66 of CBC20 which was reproducibly under-represented or absent (Additional file 6: Table S2). However, individual digestion with trypsin of either CBC20 or CBC80 from the same sample aroused all predicted peptides. Remarkably, the peptide 37–66 in the crystal structure of the CBC [29] corresponded to the interface between CBC20 and CBC80, which could be the site of interaction between ABX464 and the CBC [30] (Additional file 5: Figure S4 a and b). The same results were obtained with ABX464-*N-*glucuronide, a more soluble derivative of ABX464 that is produced as unique metabolite using human hepatocytes (see below).

However, neither ABX464 nor its metabolite ABX464-*N-*glucuronide affected the binding of CBC complex (Figure 4e) to capped RNA probe in a gel mobility shift assay [31]. While the complex between CBC and capped RNA was competed by the m^7^GpppG, no competition was observed with ABX464 or ABX464-*N-*glucuronide at any of the concentrations tested, confirming that ABX464 did not interact with the cap binding site of CBC20 (Figure 4e). Our results support the idea that ABX464 bound directly to CBC to specifically prevent Rev-mediated export of viral RNA without interfering with cap binding or export of cellular transcripts.

### Efficacy of ABX464 in humanized mouse models

**Figure 5:**
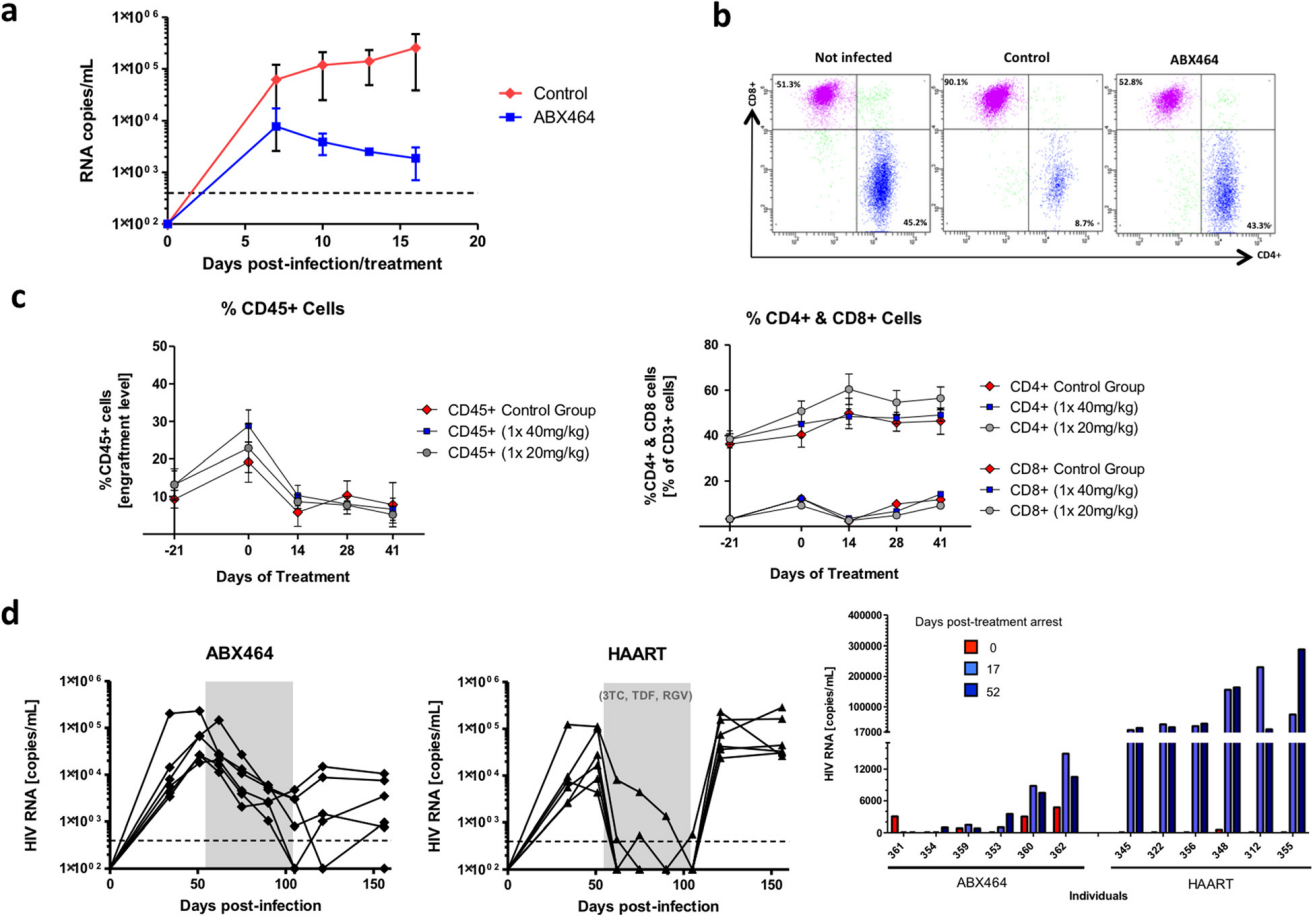
**Efficacy of ABX464 to inhibit viral replication in humanized mice. a** Reconstituted SCID mice were infected with JRCSF HIV-1 strain by intraperitoneal injection. The control group received labrafil and 5% DMSO by gavage (n = 15) and treated group 20 mg/kg b.i.d of ABX464 in labrafil and 5% DMSO (n = 14) for 15 days. Two independent experiments were performed with 5 and 10 reconstituted mice for each group. Viral load was assessed by measuring viral RNA using the Amplicor HIV-1 Monitor from Roche (limits of detection are delimited by interrupted lines). **b** FACS analysis was performed on cells recovered by peritoneal wash at day 15 post-treatment to assess the CD8/CD4 ratio. **c** Engrafted NSG humanized mice were treated by oral gavage with ABX464 at either 20 mg or 40 mg/kg once a day for 30 days and indicated lymphocyte populations were monitored by FACS analysis. **d** NSG humanized mice were infected with the YU2 HIV-1 virus and treated either by oral gavage with ABX464 at 40 mg/kg once a day for 30 days or by HAART (3TC-Tenofovir-Raltegravir) (grey boxes of left and middle panels). Right panel represents viral loads after treatment cessation. For HAART, food pellets were made by mixing 2.5 g of 3TC and TDF each, and 5 g of RTV with 5 kg of ground protein-rich, vitamin-fortified food (Nafag 3432, Provimi Kliba AG, Switzerland) which was subsequently formed to food pellets and sterilized by gamma-irradiation with 25 kGy. Viral load was assessed by measuring viral RNA using the Amplicor HIV-1 Monitor from Roche (limits of detection are delimited by interrupted lines).

Humanized mice reconstituted with human lymphoid cells provide rapid, reliable, reproducible experimental systems for testing the efficacy of ABX464 *in vivo* [32,33]. In the initial setting, SCID mice were reconstituted with PBMCs and then infected with the HIV-1 strain JR-CSF [33,34]. Mice were treated twice a day (b.i.d) for 15 days by oral gavage with 20 mg/kg of ABX464. Measures of viral RNA showed that the oral treatment with ABX464 was able to significantly reduce the viral load over a period of 15 days of treatment (Figure 5a). FACS analysis of blood samples showed that treatment with ABX464 prevented depletion of CD4+ cells following infection of reconstituted mice and thereby restored the CD8+/CD4+ ratio back to that of non-infected mice (Figure 5b).

To test the long term effect of ABX464 on the immune system and viral replication in infected humanized mice (hu mouse), newborn NOG mice were transplanted with CD34+ haematopoietic progenitor cells isolated from the umbilical cord blood [35]. This hu mouse model has previously been shown to be accurate for exploring the antiviral potency of new compounds targeting the latent HIV reservoirs [35]. Treatment of NOG hu mice for one month with 20 mg/kg or 40 mg/kg of ABX464 neither altered engraftment values of CD45+ cells nor the ratio of CD8+/CD4+ compared to controls without treatment (Figure 5c). In this study NOG hu mice were infected with the YU2 HIV-1 virus and fed daily for 30 days with 40 mg/kg of ABX464 (Figure 5d left panel) or with HAART (3TC-Tenofovir-Raltegravir) (Figure 5d middle panel) and viral loads were measured as explicated before. Most significantly, unlike HAART, ABX464 leads to a sustained reduction of viral levels in humanized mice after treatment cessation (Figure 5d right panel). Compared to HAART, ABX464 treatment induced a slower kinetic of viral load reduction (Figure 5d left panel). Whereas HAART was very efficient at reducing the viral load to undetectable level after 2–3 weeks of treatment in all infected mice (Figure 5d middle panel), with ABX464 only 2 out of 6 mice had undetectable levels of viral load, 2 mice were under 1000 copies and 2 mice are still have a viral load higher than 6000 copies after 1 month of treatment (Figure 5d left panel). While we cannot exclude the possibility that the slow kinetics of viral reduction could be inherent to ABX464’s mode of action, it is also possible that in this mouse model ABX464 used as monotherapy, is inefficient at reducing the viral load to undetectable levels. For instance mice put on 3TC treatment alone for 42 days all had a viral load above ABX464 (unpublished data). The most striking result is when the HAART treatment was stopped, 6 out 6 mice had a high viral load of above 17,000 RNA copies, with some reaching 200,000 copies (Figure 5d right panel). In contrast, only 2 mice out of 6 treated with ABX464 demonstrated any rebound and that was lower than the rebound after HAART (Figure 5d right panel). Furthermore, these two mice had a viral load of 1,000 copies when we stopped the ABX464 treatment, meaning that ABX464 had controlled most of the cells contributing to viral rebound, at least during the 52 days of treatment cessation. Moreover, 2 mice had undetectable levels of viral load during the whole period of ABX464 cessation and two mice had low viral loads of under 1,000 copies (Figure 5d right panel). These results mean that even though we can detect viruses in the blood, most infected cells in the body that could contribute to viral rebound, mostly latently infected resting CD4+ T cells and macrophages with integrated viral DNA [36,37], are not producing new viruses after ABX464 treatment cessation.

### ABX464 is readily metabolized and is non-toxic

Pharmacokinetic studies showed that ABX464 was rapidly absorbed, reaching a maximal plasma concentration within a few hours of dosing in mice (Additional file 7: Figure S5a). These results showed that the long lasting effect in hu mice was due to a reduction of infected cells and not due to a sustained accumulation of ABX464 in the mouse. We tested the potential formation of ABX464 metabolites using cryopreserved hepatocyte primary cultures from various species. The only ABX464 metabolite detected upon incubation with human hepatocytes was the ABX464-*N*-glucuronide (Figure 1a, Additional file 8: Figure S6 a and b). This metabolite was also formed by mouse and non-human primates, but not by rat, minipig or dog hepatocytes (Additional file 8: Figure S6 a and b). In the mouse at T_max_ there was two times more ABX464*-N*-glucuronide than ABX464 (Additional file 7: Figure S5a). Interestingly, *in vitro* ABX464-*N*-glucuronide was as efficient as ABX464 in inhibiting replication of virus strain YU2 in primary macrophages, without inducing any toxicity (data not shown). Thus both ABX464 and ABX464-*N*-glucuronide could be expected to induce inhibition of viral replication in humans. However, neither glucuronide nor ABX464 were still detectable after 24 hours post-treatment in mice (Additional file 7: Figure S5b right panel). Moreover, cumulating dosing of ABX464 did not sustain the presence of neither ABX464 nor ABX464-*N*-glucuronide over 6 days after treatment cessation (Additional file 7: Figure S5b left panel). Therefore, we concluded that the long lasting effect, at least in mice, cannot be attributed to glucuronide accumulation.

Furthermore, pharmacokinetic and toxicology studies performed in non-human primates showed that ABX464 was rapidly absorbed with a maximal plasma concentration reached after 2 to 4 hours in marmoset monkeys (Additional file 7: Figure S5c). ABX464-*N*-glucuronide was formed rapidly with comparable T_max_ values but C_max_ and AUC values were much higher for the metabolite than for the parent compound in non-human primates (Additional file 7: Figure S5c).

In rat, the no observed adverse effect level (NOAEL) of ABX464 was achieved at a dose of 55 mg/kg b.i.d (Additional file 2: Table S1). In non-human primates, the main target organ of ABX464 toxicity was found to be the gastro-intestinal tract. The NOAEL was considered to be 250 mg/kg b.i.d (Additional file 2: Table S1). Rough estimate of the concentration in the animal indicate that these NOEL dose are 50 times above the efficient dose required to inhibit viral replication *in vitro.*

## Discussion

In our attempt to identify new more potent and non toxic HIV RNA splicing modulators inspired by the structure of IDC16, we present ABX464 which we show targets Rev function and therefore is susceptible to alter the viral expression in the reservoirs. The Rev protein facilitates the transport of unspliced and singly-spliced RNA to the cytoplasm in infected host cells by binding to RRE and competing with the major process of cellular mRNA export [11,27,38]. This Rev’s essential role in HIV replication, its mode of action and specific interactions with its target RNA and cellular proteins enhance to the attractiveness of Rev as a target. Despite the fact that, a variety of approaches targeting Rev function, (including gene therapy [39-41]) have been developed to inhibit HIV-1 replication in cells cultured *in vitro* [42-49], no therapies are clinically available based on this mechanism. Many molecules selected this way have shown considerable toxicity in cells or have failed to specifically inhibit HIV replication [42,43,45].

The finding that ABX464 binds to and stabilizes the CBC complex (Figure 4 and Additional file 5: Figure S4), together with the recent demonstration that Rev protein specifies the viral RNA pathway by competing with TREX complex [27], suggest that ABX464 acts as an agonist to favour the normal pathway of export of spliced mRNAs. Consistent with this suggestion, we found that ABX464 enhances the expression of spliced viral RNA, while reducing the expression of unspliced RNA without having any effect on cellular splicing (Figure 3).

Previous studies have shown that the presence of the CBC complex on the 5′ RNA cap favours the recruitment of TREX on the opened DNA at the transcription site for efficient processing and export of the mRNP [50,51], competition between Rev and TREX complex may occur at the transcription site. Our data together with recent observations using the MS2 system which allows for visualization of events at the transcription site and export [24], further confirm the hypothesis that Rev and TREX compete with one another. The recruitment of export factors to the site of transcription represents a mechanism by which specific RNAs can be exported to the cytoplasm by alternative mechanisms. If unspliced viral RNAs are committed, to the CRM1 dependent export, by Rev [24], TREX-specific transport of multispliced will be diminished [27,50,52]. Whether this competition mechanism involves direct interaction between Rev and CBC complex has been shown recently; Rev interacted with the CBC80 subunit of the CBC complex and inhibited the recruitment of the TREX complex [27]. ABX464 stabilizes the binding of CBC at the cap structure and thus prevents Rev from seizing control of the TREX that binds to the cap-proximal region and determines the RNA export pathway and splicing [50-52].

Current therapies specifically target individual viral enzymatic activities and this confers relatively little risk of toxic effects to the host but the risk of developing resistant viruses is high [53]. ABX464, on the other hand, exploits a smaller windows of specificity by interfering with Rev-mediated export pathway though direct binding to the CBC complex. ABX464 is unlikely to induce the development of resistance since it targets a cellular, rather than a viral, component, (the CBC complex), ABX464 is also expected be active on different HIV clades and mutants viruses. Results presented here are consistent with this expectation (Figure 2). ABX464 is globally not toxic to infected cells because it has little effect on the export of bulk mRNAs and did not alter splicing of cellular genes nor the production of snRNAs nor of histones mRNAs whose biogenesis are dependent on the CBC complex [26]. By preventing Rev function, ABX464 restores the normal pathway of global RNA biogenesis that is disturbed by Rev expression in infected cells (with integrated proviral genome). ABX464 outperforms known drugs that target Rev in toxicological studies in all these ways and could be used in combination with current treatments or as a simpler and more effective replacement.

ABX464 reduces the viral load over a period of 30 days of treatment (Figure 5d left panel) but more importantly, the viral load remains low for at least 52 days after treatment termination (Figure 5d right panel). In contrast, rebound up to levels comparable to the initial infection is seen in the HAART group (Figure 5d right panel). ABX464 is thus the first anti-HIV drug able to suppress viral load sustainably after treatment arrest. The long lasting effect of ABX464 in mice could be explained by the finding that ABX464 prevents HIV replication in macrophages. The HIV-1 infected macrophages are of critical importance in the pathogenesis of HIV because macrophages have the ability to cross the blood-tissue barrier and deliver the virus to all tissues and organs, including the brain [37]. In addition, unlike T lymphocytes that are depleted by viral infection, macrophages are relatively less prone to the cytopathic effect of the virus and infected macrophages harbour and produce the virus for a longer period of time. Macrophages treated by ABX464 are unable to produce viral particles to transmit HIV-1 from macrophage to CD4+ T cells [36]. As macrophages act as the antigen presenting cells and present processed pathogen antigen peptides to the CD4+ T cells via MHC II pathway [54], one would assume that viral proteins produced from multispliced transcripts could serve as antigens to boost the immune anti-viral response. Given that the expression of these multispliced viral RNA will depend on the chromatin organization of the proviral DNA in infected macrophages [55,56] and the cross talk between chromatin and the splicing machinery [57-59], one would speculate that ABX464 acting on the CBC complex at the transcription site, might set up a chromatin structure favoring the splicing of the primary transcript and that this could thereby set up the long lasting effect of the drug.

## Conclusions

In this report we present ABX464 first trials. Interestingly, this drug presents a new mechanism of action for HIV treatment and improves current therapies, hampering appearance of virus resistance. More importantly, it leads to a long lasting control of the viral levels. We propose that ABX464 could be used in combination with current drugs or as a simpler and more effective replacement.

## Methods

### Ethics statement

All animal procedures were conducted in strict adherence with the European Community Council Directive of 24 November 1986 (86–609/EEC) and approved by the cantonal veterinary office Zurich (#26/2011).

The procurement and use of CD34+ cells from human cord blood was approved by the Cantonal Ethical Committee of Zurich (EK-1103). All adult subjects provided written informed consent. Animal care and experimental protocols were in accordance with the “Swiss Ethical Principles and Guidelines for Experiments on Animals”, and approved by the Veterinary office of the Canton of Zurich, permit 26/2011. Manipulations of mice were in accordance with the regulations of the Veterinary office of the Canton of Zurich. (http://www.veta.zh.ch/internet/gesundheitsdirektion/veta/de/home.html).

### Drug dilution and handling

A 50 mM stock solution of each tested compound was made in 100% DMSO and stored between −20°C and +4°C. An intermediate solution at 500 μM was prepared in cell culture medium (RPMI) complemented with 10% fetal bovine serum. This solution was agitated for one hour at 37°C on a Thermomixer at 1000 rotations per minute and then stored over-night at +4°C. The following day, the solution was subjected to centrifugation (16,000 rpm) for 5 minutes and the supernatant was used to prepare the various dilutions in cell culture media complemented with 10% fetal bovine serum, antibiotics (penicillin and streptomycin) and IL-2 cytokine that were added to PBMCs.

### Cell culture cytotoxicity evaluation and infection

Buffy coats from HIV-negative individuals were obtained from the local blood donation center in Zurich, Switzerland (http://www.blutspendezurich.ch/) and Centre de transfusion sanguine Montpellier. Human peripheral blood mononuclear cells (PBMCs) were isolated by Ficoll (Axis-Shield PoC AS) gradient centrifugation. The cells were grown at 37°C, 5% CO_2_ to a density of 1.5×10^6^ cells/ml in RPMI Glutamax medium (Life Technologies) supplemented with 10% fetal calf serum (FCS) (Thermo Fischer), 1,000 U/mL of IL2 (Peprotech) and 5 μg/mL of PHA (Roche) for activation. Three days later, cells were pooled and resuspended to a density of 1.5×10^6^ cells/ml in RPMI Glutamax medium supplemented with 10% fetal calf serum (FCS) and 1,000 U/ml of IL-2 for infection. HIV-1 infection has been performed with 10 μg of Ada-M R5 HIV strain per ml of cells for 4 hours. Cells were then centrifuged and resuspended to a density of 1.5×10^6^ cells/ml in medium supplemented with diluted drug solubilized in DMSO (Sigma) according to a final 0.05% DMSO concentration. Cells were treated for 6 days with a partial medium change at day 3. From cell culture supernatants, HIV p24 titration was performed by ELISA with Ingen Innotest kit (Ingen) according to manufacturer’s instructions.

To evaluate the cytoxicity of different compounds we used the same protocol as above to seed the PBMCs in a final volume of 100 μl without adding the virus. After an incubation for 6 days at 37 C, a cell proliferation assay has been performed. It is based on the conversion of 5-(3-carboxymethoxyphenyl)-2-(4,5-dimethylthiazolyl)-3-(4-sulfophenyl)tetrazolium (MTS) to the coloured product formazan in the presence of phenazine methosulfate. The colorimetric conversion was proportional to the number of viable cells in culture. The CellTiter 96® AQ_ueous_ One Solution (Promega) has been added to the assay wells (20 μl/well). Cells were incubated 4 hours at 37°C and then absorbance is measured at 492 nm. Mean and standard deviation of absorbance values for triplicates were calculated. The cell viability percentage of treated cells was determined by comparison between treated cells absorbance value and untreated cells. The cytotoxicity percentage was inversely deducted. Finally, the TC_50_ was calculated by regression to determine the compound concentration responsible for 50% of cytotoxicity. To generate monocyte derived macrophages (MDMs), monocytes were isolated using CD14 microbeads (Miltenyi) and cultured in X-VIVO10 medium (Lonza) supplemented with GM-CSF 1,000 U/ml and M-CSF 100 ng/ml for 6 days. Monocytes were seeded at a cell count of 50,000 cells per well in a 96 well plate. After 6 days medium was replaced with X-VIVO10 w/o cytokines. After 2 days macrophages were treated with ABX464 or ABX464-*N-*Glucuronide o/n and next day infected with Yu-2 virus for 6 hours, washed with PBS and cultured in medium containing the compounds for 12 days. Supernatant for p24 ELISA was collected 2 times a week.

HeLa cells from ATCC were cultivated at 37°C, 5% CO_2_ in DMEM Glutamax medium (Life Technologies) supplemented with 10% fetal calf serum (FCS) (Thermo Fischer). pΔPSP (2 μg per 400,000 cells) transient transfection was performed by JetPEI reagent (PolyPlus) according to manufacturer’s instruction.

Jurkat CCR5 cells were cultivated at 37°C, 5% CO_2_ in RPMI Glutamax medium (Life Technologies) supplemented with 10% fetal calf serum (FCS) (Thermo Fischer) at 1.5×10^6^ cells/ml. HIV-1 infection has been performed with 30 ng of Ada-M R5 HIV strain per ml of cells for 4 hours. Cells were then centrifuged and resuspended to a density of 1.5×10^6^ cells/mL, plated in 6 multiwell plates (2 ml/well) supplemented with 2 ml diluted DMSO solubilized drug (Sigma) according to a final 0.1% DMSO concentration. Cells were treated for 3 days. Total RNA was extracted with the Machery-Nagel Nucleospin Triprep kit. RNA was reverse transcribed utilizing a random hexamer primer. Quantitative PCR (qPCR) analysis of the viral transcripts was obtained with the following primer pairs [RefSeq: M19921]: for all viral RNAs GGCCTGCTGTAAGGGAAAG (8824–8842) and CTTGTGCCTGGCTAGAAGC (8965–8947), for unspliced RNAs CTGAAGCGCGCACGGCAA (706–723) and GAGATGGGTGCGAGAGCGTC (806–787) and for multispliced RNAs GACTCATCAAGTTTCTCTATCAAA (6018–6041) and ACCACCGCTTGAGAGACT (8534–8517). Each sample was normalized by TATA box binding protein (TBP) [RefSeq: NM003194] transcripts detected with the primers GTTTCTTGGCGTGTGAAGATAACC (139–162) and GAAACCCTTGCGCTGGAACTCGT (252–230). qPCR was performed utilizing a Roche LightCycler real-time PCR system and SYBR green dye, assays were carried out in triplicate and analyzed with the LightCycler software.

U937 cells were grown at 37°C, 5% CO_2_ in RPMI Glutamax medium (Life Technologies) supplemented with 10% fetal calf serum (FCS) (Thermo Fischer) at 1×10^6^ cells/ml. Cells were pre-treated 48 hours with 10 μM of molecule, then, single round infection has been performed with 150 ng of P24 of a NL4.3 R^−^E^−^LUC-VSVg production, per ml of cells in 6 multiwell plates (2 ml/well) with or without ABX464 or AZT at 10 μM. Cells were harvested at 24 hours and 48 hours post-infection, wash once with PBS and one tenth of cells were lysed with 200 μl of Passive Lysis Buffer (Promega). Protein quantification was assessed by Bradford assay performed by adding 200 μl of Bradford solution to 10 μl of cell lysate (BioRad). After a brief incubation, the absorbance was measured at 595 nm. Protein concentrations are estimated by reporting to a BSA standard curve. In parallel, a luciferase assay (Promega) was performed by adding 70 μl of luciferin in 20 μl of cell lysate. Luminescence of each point was measured and normalized by the protein concentration before being reported to the control.

HeLa 128*MS2-GFP cells (kind gift from E.Bertrand, IGMM Montpellier, France) were maintained in DMEM Glutamax medium (Life Technologies) containing 10% fetal bovine serum (Hyclone), penicillin/streptomycin (10 U/ml) in a humidified 5% CO2 incubator at 37°C. Cells were transfected with pSG-FlagRev plasmid (kind gift from P.Jalinot, ENS Lyon, France) for 24 hours with JetPEI reagent (PolyPlus) according to manufacturer’s instruction.

### Selections for drug resistance and studies on resistant viruses

Selections for resistance against various antiretroviral compounds (ARVs) were performed as previously described [60]. Briefly, HIV-1 infected cultures were grown in the presence of the various ARVs tested beginning with drug concentrations that were at or below the IC50 in each case, including ABX464. Drug concentrations were gradually increased and possible resistance-related mutations in relevant regions of the HIV genome, such as the reverse transcriptase open reading frame, were identified by sequencing. In the case of ABX464, it was not possible to increase drug concentration above the IC50 because no evidence of drug resistance was obtained.

Viruses of different subtypes or clades and viruses containing the M184V and K65R resistance-associated mutations in reverse transcriptase were generated as previously described [61,62].

### Immunofluorescence analysis

Cells plated on cover slips were fixed for 10 min in 3.7% formaldehyde (in PBS) followed by a 2 min permeabilization with 0.1% Triton X-100 (in PBS) and incubation in PBS containing 0.1% bovine serum albumin. Rev protein was revealed using antibody against HIV-1 Rev protein (Santa Cruz) and nuclei were stained using Hoechst 33342 (Sigma-Aldrich). Cells were washed in PBS mounted with DAKO mounting medium and observed under the fluorescence microscope.

Cell imaging was performed with a Leica DM6000 (Leica, Wetzlae, Germany) with PL APO grade oil × 63 objective. Images were captured with a Coolsnap HQ2 camera (Roper Scientific Inc.) driven by Metamorph (Molecular Devices) and processed using FiJi software and GFP fluorenscence intensity was determined using the measure module of FiJi.

Image acquisition and image analysis were performed on workstations of the Montpellier RIO Imaginig facility of Plateau MRI CRBM Optique.

### HIV RNA capture and sequencing

HIV RNA was captured on a NimbleGen SeqCap EZ Developer Library following the manufacturer’s protocols (Roche/NimbleGen). EZ Oligo pool was made against Ada-M R5 HIV strain. Total RNA was extracted from PBMCs samples using TRIZOL reagent (Invitrogen) following the manufacturer’s protocol. The RNA concentrations and purity were determined using the RNA 6000 Bioanalyzer kit (Agilent). For cDNA synthesis, 5 μg of total RNA was treated with DNase I (Invitrogen) and then utilized as a template for reverse transcription using the Verso cDNA kit with a blend of random hexamers and anchored oligo-dT (Thermo Scientific), according to the manufacturer’s instructions. Libraries for sequencing were prepared and sequenced using 454 pyrosequencing (according to GS junior method manual).

### Generation of humanized mouse models and infection

SCID mice were reconstituted with fresh human PBMCs for two weeks and the reconstitution rates were estimated by human IgG titration as previously described [32,33]. Reconstituted SCID mice were infected with JRCSF HIV-1 strain by intraperitoneal injection. Control group received labrafil and 5% DMSO by gavage (n = 15) and the treated group received 20 mg/kg b.i.d of ABX464 in labrafil and 5% DMSO (n = 14) for 15 days.

NOD.scid.IL2R −/− (NSG) mice were bred and maintained in individual ventilated cages and were fed autoclaved food and water. Mice with a human immune system (NSG-HIS) were generated as described [35]. Briefly, newborn (<5 days old) NSG mice received sub-lethal (1Gy) total body irradiation with a Cs source, and then received 2× 10^5^ transduced or untransduced CD34^+^ human HSCs using a 50 μl Hamilton syringe via the intrahepatic (i.h.) route. Mice were infected intraperitoneally (i.p.) with HIV YU-2, 1x10^6^ TCID50 per mouse. All manipulations of NSG-HIS mice were performed under laminar flow. Gavage of mice was performed daily with a stainless steel gavage needle (Straight 22 Gauge, 1,4inch in length). ABX464 was dissolved in DMSO (Sigma), and then diluted to 5% or less according to the dose required in a suitable vehicle (Labrafil; COOPER INDUSTRIE). Mice did not receive more than 150 μl in volume per day. Mice were monitored three times a week for symptoms or signs of adverse events, according to a standard score sheet. HIV RNA plasma levels were measured by RT-PCR (AmpliPrep/COBAS TaqMan HIV-1 Test, Roche) at various times after infection.

### HIV virus stock

Viral stocks were obtained by polyethylenimine (PEI)-mediated transfection (Polysciences) of 293 T cells with a pYU-2 (R5 tropic) plasmid provided through the NIH AIDS Research and Reference Reagent Program. 48 hours after transfection, the virus was harvested, filtered (0.45 μm), and frozen at −80°C. Viral titers were determined as previously described [63]. Briefly, TCID50 (tissue culture infectious dose 50%) was determined by infecting human CD8+ T-cell-depleted peripheral blood mononuclear cells (PBMCs) from three donors which were stimulated by addition of IL-2, PHA and anti-CD3 beads (Life Technologies).

### Flow cytometry

Cell suspensions were labeled with anti-human monoclonal antibodies (mAb) targeting the following cell-surface markers: CD45-FITC, CD3-PE, CD4-Pe Cy7, CD8-BV421 and CD19-APC (all from Biolegend). Washing and reagent dilutions were done with FACS buffer (PBS containing 2% fetal calf serum and 0.05% sodium azide (NaN3). All acquisitions were performed on a Cyan ADP (Beckman Coulter) flow cytometer. Data were analysed with FlowJo software (Ashland, OR). Cellular debris and dead cells were excluded by their light-scattering characteristics.

## Additional files

Additional file 1: Figure S1. Sequencing of HIV-1 genome after ABX464 treatment. a. HIV-1 strain YU2 was used to infect macrophages from 4 different donors (D1, D2, D3 and D4) and viruses from the supernatant of untreated (1–4) and treated (5–8) samples were harvested 8 days post-infection (pi) and subjected to deep sequencing using 454 pyrosequencing (according to GS junior method manual). Upper panel schematic representation of different ORFs encoded by HIV-1 genome. Position of all detected mutations/polymorphisms (POS). Corresponding codons altered by the mutations at each position (CODON REF/ALT) and expected amino acid changes (AA ref/Alt). Reference nucleotide (REF) the corresponding mutated nucleotide (ALT) at each position. In blue are the positions that were found to be mutated (the numbers indicate the frequency of each mutation). In red are the positions that are unaffected. b. Viruses from treated sample 5 in (a), was used to infect PBMCs from donor D3 and D4 and viruses from the supernatant of untreated (9 and 10) and treated (11, 12) samples were harvested 6 days pi and subjected to deep sequencing using 454 pyrosequencing (according to GS junior method manual). Additional file 2: Table S1. Tabular overview of toxicology studies. Additional file 3: Figure S2. Effects of ABX464 on HIV RNA splicing and viral DNA integration. a. schematic representation of HIV-1 genome. b. ABX464 fails to inhibit splicing in HeLa cells transfected by p△PSP plasmid. HeLa cells transfected with the p△PSP construct were either untreated (DMSO) or treated with 5 μM or 10 μM of compound ABX464. Multiply spliced products of HIV-1 RNA were amplified by RT-PCR according to Bakkour et al. [13]. The PCR products were analyzed on a LabChip HT DNA assay station (Caliper) for quantitation and sizing (according to manufacturer’s instructions). Nomenclature of the RT-PCR products on the right of the panel is according to Bakkour et al. [13]. c. The intensity of each indicated band amplified in (b) was used to rigorously quantify the changes in splicing products. Values are the average of three independent experiments for untreated samples (DMSO) (white), treated with 5 μM (grey) or 10 μM (black) and the level of expression is normalized with that of total amplified signals. The value of untreated samples is equal to 1. d. ABX464 enhanced expression of mutispliced viral RNA in infected cells. The values of total RNA, unspliced and multispliced RNA in the untreated sample are set to 100% (white) and the values of the same RNAs in treated sample (grey) were compared to these values (left panel). The ratio between multispliced and unspliced RNA in untreated (white) and untreated sample (grey) are shown (right panel). e. ABX464 acts after integration of proviral DNA. U937 cells were pre-treated 48 hours with 10 μM of molecules of interest, then, single round infection has been performed with NL4.3 R^−^E^−^LUC-VSVg with or without ABX464 or AZT at 10 μM as described in methods. Additional file 4: Figure S3. GFP expression of HeLa 128*MS2-GFP cells expressing HIV reporter. Comparison between untreated (DMSO) or ABX464 treated cells. Additional file 5: Figure S4. ABX464 interaction with CBC complex. a. Purified recombinant CBC20 and CBC80 proteins were incubated with the indicated concentrations of ABX464-*N*-glucuronide and they were either untreated (lanes 1–3), treated during 15 min (lanes 4–6) or 30 min (lanes 7–9) with UV light. Proteins were analyzed by SDS-PAGE and stained with Coumassie blue. ABX464 and ABX464-*N*-glucuronide promotes UV crosslinking of CBC20 and CBC80. b. Recombinant human CBC was incubated with ABX464-*N*-glucuronide and treated with UV for 15 min. After crosslinking the proteins were resolved in SDS-PAGE and stained with Coomassie blue (left panel). Stained proteins (Band 4, Band 5 and Band 7) were digested with trypsin and analysed by mass spectrometry (details of mass spectrometry analysis of two independent experiments are in Additional file 6: Table S2). Right panel shows the Ribbon representation of the CBC complex (CBC20 in brown and CBC80 in purple) with the position of the cap RNA (in blue/red) on CBC20 and the putative peptide of CBC20 that interacts with ABX464 (yellow). Additional file 6: Table S2. Mass spectrometry analysis of CBC complex and ABX464 interaction. Additional file 7: Figure S5. ABX464 pharmacokinetics parameters. a. Main plasma pharmacokinetic parameters of ABX464 and ABX464-*N*-glucuronide in the mouse after an administration of 40 mg/kg of ABX464 by the oral route. b. Plasma pharmacokinetic parameters of ABX464 and ABX464-*N*-glucuronide in the mouse after oral administration. Female OF1 mice received 40 mg/kg of an ABX464 suspension in Labrafil - DMSO 5% per os, once a day during 28 days. Blood was sampled at day 0, 7, 14, 21 and 28 (left panel) or 0, 0.5, 2, 4, 6, 9 and 24 hours after gavage at day 0 (right panel) (5 animals per time point). After day 28 the gavage was stopped in left panel (arrow shows end of the treatment). After 6 days of stop of the treatment (left panel) or 24 hours post treatment (right panel), neither ABX464 nor ABX464-*N*-glucuronide were still detectable. ABX464 and ABX464-*N*-glucuronide concentrations were quantified in plasma by LC-MS. c. Plasma pharmacokinetic parameters of ABX464 in in the marmoset monkey after one oral administration of 750 mg/kg of ABX464. Additional file 8: Figure S6. ABX464 *in vivo* metabolism. a. *In vitro* metabolism profiling of ABX464 in cryopreserved hepatocytes primary cultures from various species. Hepatocytes were incubated with 10 μM ABX464 for 120 minutes at 37°C. Aliquots from each supernatant culture were analyzed by LC-MS/MS to identify potential ABX464 metabolites. The number of peaks present in the chromatogram is indicated for each metabolite in the corresponding species. b. Drawing and formula of ABX464 metabolites.
