# Supplementary material for: Long lasting control of viral rebound with a new drug ABX464 targeting Rev – mediated viral RNA biogenesis
Source: Retrovirology. 2015 Apr 9;12:30. doi: 10.1186/s12977-015-0159-3 (PMC4422473; doi:10.1186/s12977-015-0159-3)
Supplement: Additional file 2: Table S1. — Tabular overview of toxicology studies. [file 12977_2015_159_MOESM2_ESM.pdf]

Study type	Species / Strain	Dose-level	Route	Endpoints evaluation	Significant findings
MLA assay	L5178Y mouse lymphoma cells	0.24-50 µg/mL	*In vitro*	• Mutant frequency	• No statistically or biologically significant increases in the mutant frequency, without as well as with S9 metabolic activation.
Ames' test	Histidine-dependent strains of *Salmonella typhimurium* (TA98, TA100, TA1535, TA1537 and TA102)	Plate incorporation method<br>5.1-500 µg/plate<br>Pre-incubation method<br>0.53-10 µg/plate	*In vitro*	• Revertant frequency	• No statistically or biologically significant increases in the revertant frequency using both methods, without as well as with S9 metabolic activation.
Micronuclei induction in the bone marrow	Rat/Wistar<br>5/sex/group	0, 20, 55, 150, 150 mg/kg b.i.d.<br>300 mg/kg s.i.d.	*Per os*	• Number of micronucleated polychromatic eythrocytes	• No biologically significant increases in the frequency of micronucleated polychromatic erythrocytes in the rat bone marrow.
Dose Range Finding 7-day repeated-dose (no follow-up)	Rat/Wistar<br>29 males/29 females<br>5/sex/group<br>3 satellites/sex for all treated-group	0, 20, 70, 100, 150, 250 mg/kg b.i.d.	*Per os*	• **A**<br>• **B**<br>• **C**<br>• TKs	• Premature sacrifice of 1 female treated at 250 mg/kg on D6, due to poor clinical condition<br>• Lower mean body weight gain at the highest dose-levels (150 & 250 mg/kg)<br>• Hyperleukocytosis at the highest dose-levels (100 to 250 mg/kg)<br>• Lower mean total protein, albumin and globulin levels at the highest dose-levels (100 to 250 mg/kg)<br>• Dose-dependent increase of mean liver weight<br>• Decreased mean spleen weight at 250 mg/kg<br>• Dark foci in the glandular mucosa of stomach at 150 & 250 mg/kg

Study type	Species / Strain	Dose-level	Route	Endpoints evaluation	Significant findings
Dose Range Finding<br>7-day repeated-dose (no follow-up)	Rat/Wistar<br>13 males/13 females<br>5/sex/group<br>3 satellites/sex for treated-group	300 mg/kg s.i.d.	*Per os*	• **A**<br>• **B**<br>• **C**<br>• PK	• Lower mean body weight gain associated with lower food consumption<br>• Lower individual platelet count in males<br>• Lower mean total protein, albumin in either gender and globulin levels (males only)<br>• Increased mean liver weight<br>• Dark foci in the glandular mucosa of stomach
Dose Range Finding<br>7-day repeated-dose (no follow-up)	Monkey/Cynomolgus<br>1 male/1female	1000, 1500 mg/kg b.i.d.	*Per os*	• **A**<br>• **B**<br>• TKs	• Vomiting and liquid feces, probably due to large volume administered<br>• Slight body weight losses<br>• Decreases of red blood cell counts, hemoglobin, packed cell volume & mean corpuscular volume<br>• Increased APTT and prothrombin time<br>• Decreased cholesterol and protein levels<br>• Increased ASAT activity.
Dose Range Finding<br>7-day repeated-dose<br>(no follow-up)	Monkey/Marmoset<br>3 males/3 females<br>1/sex/group	250 (1 day),<br>750 (1 day),<br>1000 (3 days)<br>mg/kg b.i.d.<br><br>750 mg/kg b.i.d., 1500 mg/kg s.i.d.<br>(7 days)	*Per os*	• **A**<br>• **B**<br>• **C**<br>• TKs	• Vomiting at 1000 mg/kg b.i.d., occasional, dose-related vomiting with the other dose-levels.<br>• Body weight loss for all dose-levels in all groups.<br>• Increase in spleen and kidney weights & in serum urea in 1 female at the highest dose-level.<br>• MTD = 750 mg/kg b.i.d. or 1500 mg/kg s.i.d.

Study type	Species / Strain	Dose-level	Route	Endpoints evaluation	Significant findings
Repeated-dose, 4 weeks (2 week follow-up)	Rat / Wistar<br>64 females/64 males<br>16/sex/group	20, 55, 150 mg/kg, b.i.d.<br>300 mg/kg s.i.d.	*Per os*	• **A**<br>• **B**<br>• **C**<br>• Urine analysis<br>• Histopathology<br>• TKs	• Lower terminal body weights for dose-levels ≥ 55 mg/kg b.i.d.<br>• Transient minimal to moderate histopathologic alterations in liver, Harderian gland, parotid salivary glands, stomach, thymus and pituitary gland<br>• Adverse and transient changes in the female reproductive tract (lower ovaries and uterus weights at 150 mg/kg b.i.d. and 300 mg/kg s.i.d.)<br>• All observations reversible or improved with cessation of treatment<br>• NOAEL = 55 mg/kg b.i.d.
Repeated-dose, 4 weeks (3 week follow-up)	Monkeys/ Marmoset<br>25 females/25 males<br>5/sex/group	75, 250, 750 mg/kg, b.i.d.<br>1500 mg/kg s.i.d.	*Per os*	• **A**<br>• **B**<br>• **C**<br>• Urine analysis<br>• Ophtalmology<br>• Cardiovascular investigations<br>• Histopathology<br>• TKs	• Frequent vomiting associated with SPL-464 treatment<br>• Weight loss observed in non-treated and treated groups, consolidated by vomiting in treated group<br>• Treatment stopped at day 10 for dose-levels ≥ 750 mg/kg and on day 18 for lower dose-levels due to poor clinical condition of animals, with sacrifice of 3 moribund animals at 750 mg/kg b.i.d. (day 9 and 14) and 2 moribund animals at 250 and 75 mg/kg b.i.d.(day 15).<br>• Target organ: gastro-intestinal tract (reversible)<br>• NOAEL = 250 mg/kg/day b.i.d.

Endpoints evaluation A:

- Clinical signs & mortality (daily)
- Body weight/ food consumption

Endpoints evaluation B:

- Haematology
- Serum clinical chemistry

Endpoints evaluation C:

- Necropsy & macroscopic examination
- Organ weight
- Sampling and preservation of all tissues
