# Supplementary figures and images for: Long lasting control of viral rebound with a new drug ABX464 targeting Rev – mediated viral RNA biogenesis
Source: Retrovirology. 2015 Apr 9;12:30. doi: 10.1186/s12977-015-0159-3 (PMC4422473; doi:10.1186/s12977-015-0159-3)

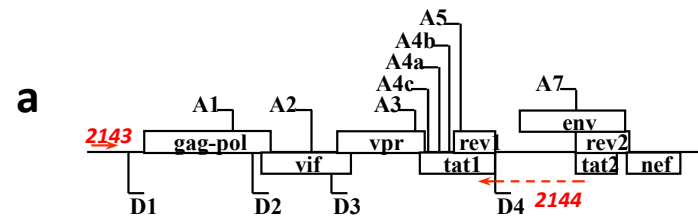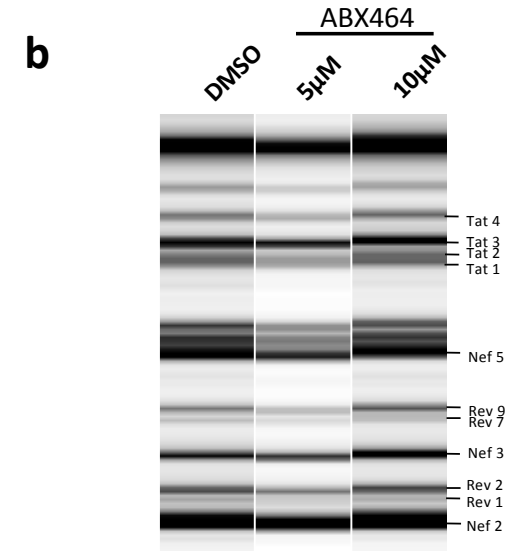

**c** HIV-1 mRNA modulation by ABX464

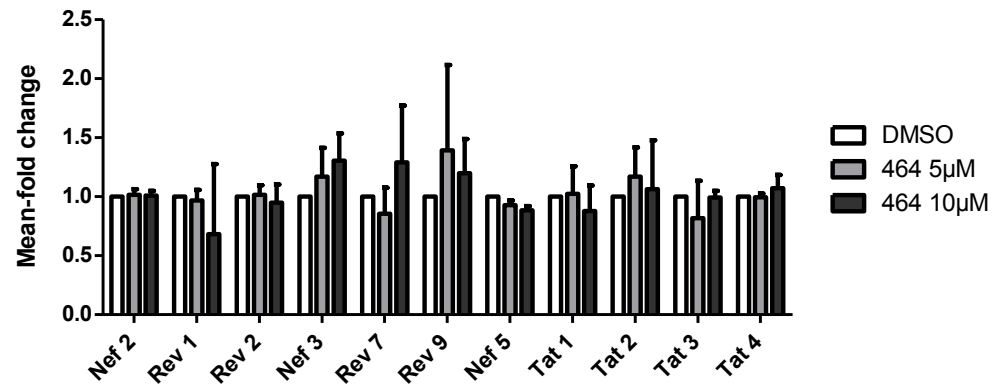

**d**

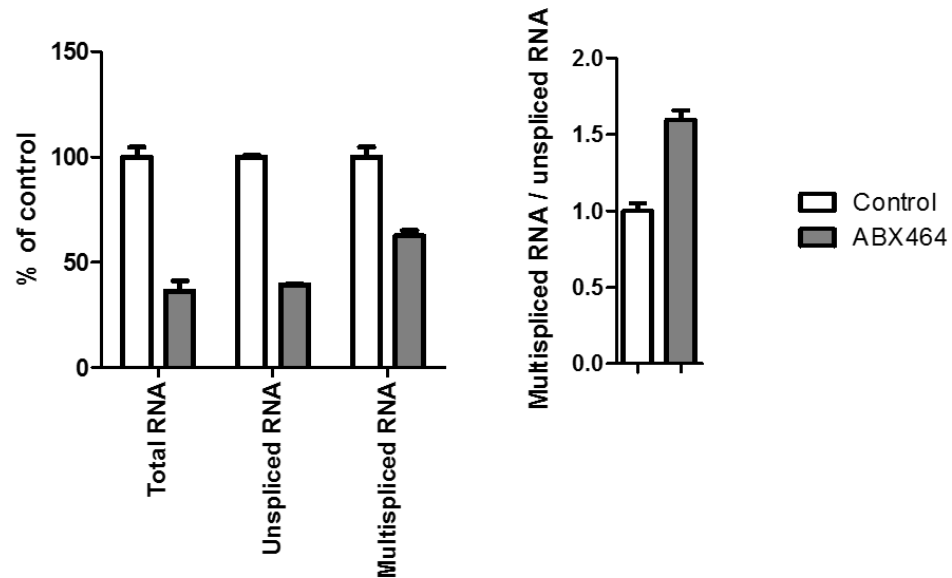

**e**

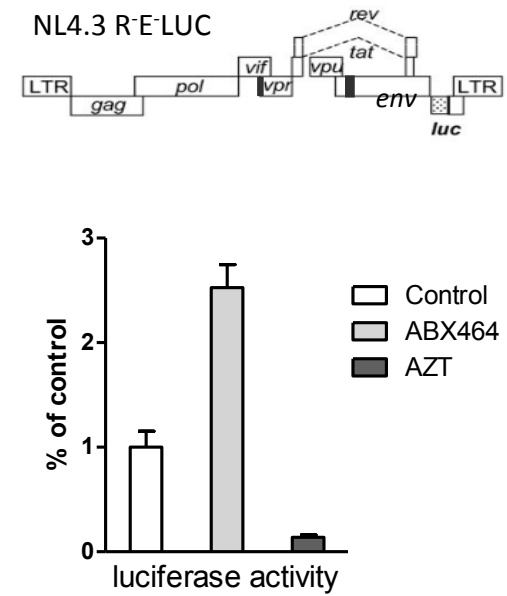

Supplement: Additional file 3: Figure S2. — Effects of ABX464 on HIV RNA splicing and viral DNA integration. a. schematic representation of HIV-1 genome. b. ABX464 fails to inhibit splicing in HeLa cells transfected by p△PSP plasmid. HeLa cells transfected with the p△PSP construct were either untreated (DMSO) or treated with 5 μM or 10 μM of compound ABX464. Multiply spliced products of HIV-1 RNA were amplified by RT-PCR according to Bakkour et al. [13]. The PCR products were analyzed on a LabChip HT DNA assay station (Caliper) for quantitation and sizing (according to manufacturer’s instructions). Nomenclature of the RT-PCR products on the right of the panel is according to Bakkour et al. [13]. c. The intensity of each indicated band amplified in (b) was used to rigorously quantify the changes in splicing products. Values are the average of three independent experiments for untreated samples (DMSO) (white), treated with 5 μM (grey) or 10 μM (black) and the level of expression is normalized with that of total amplified signals. The value of untreated samples is equal to 1. d. ABX464 enhanced expression of mutispliced viral RNA in infected cells. The values of total RNA, unspliced and multispliced RNA in the untreated sample are set to 100% (white) and the values of the same RNAs in treated sample (grey) were compared to these values (left panel). The ratio between multispliced and unspliced RNA in untreated (white) and untreated sample (grey) are shown (right panel). e. ABX464 acts after integration of proviral DNA. U937 cells were pre-treated 48 hours with 10 μM of molecules of interest, then, single round infection has been performed with NL4.3 R−E−LUC-VSVg with or without ABX464 or AZT at 10 μM as described in methods. [file 12977_2015_159_MOESM3_ESM.pdf]

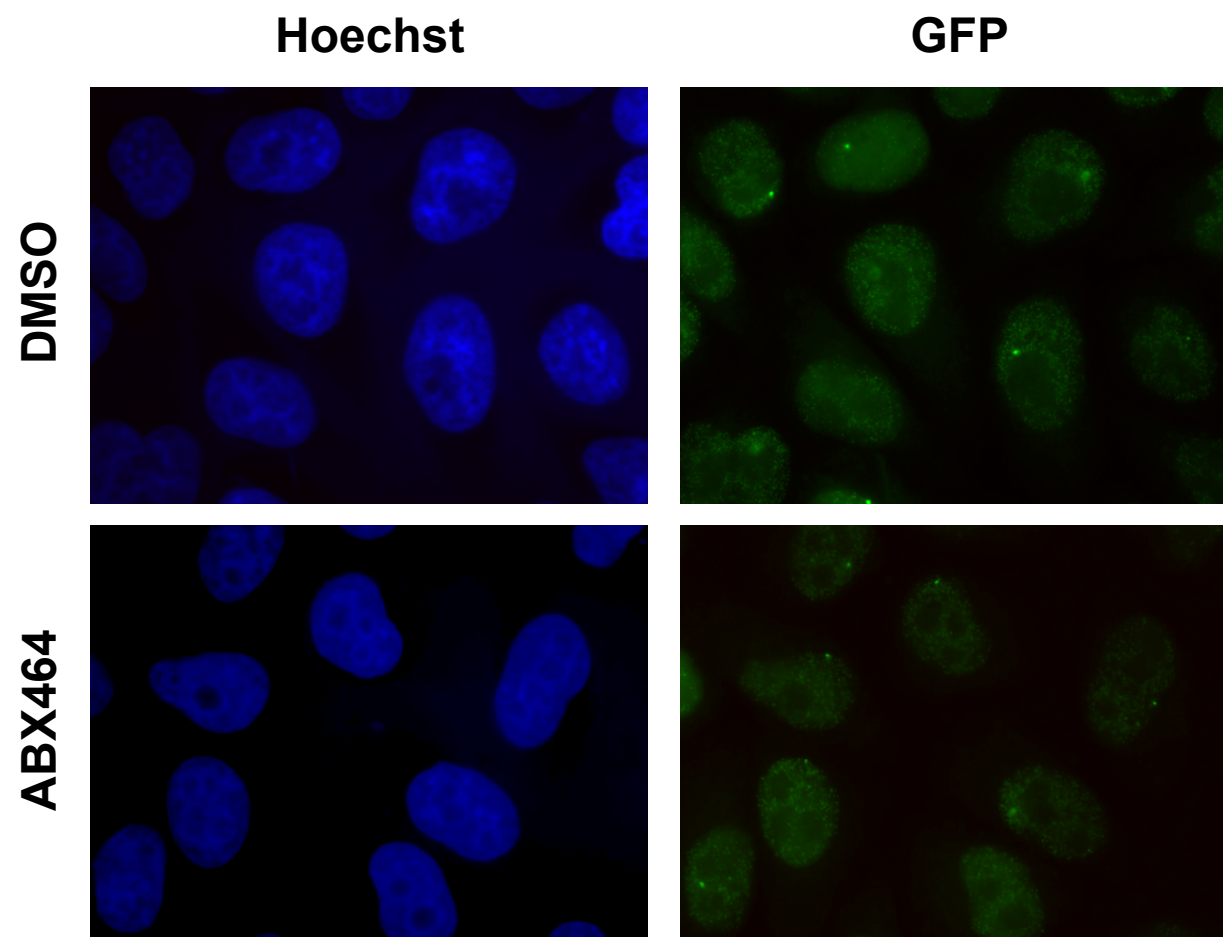

Supplement: Additional file 4: Figure S3. — GFP expression of HeLa 128*MS2-GFP cells expressing HIV reporter. Comparison between untreated (DMSO) or ABX464 treated cells. [file 12977_2015_159_MOESM4_ESM.pdf]

**a**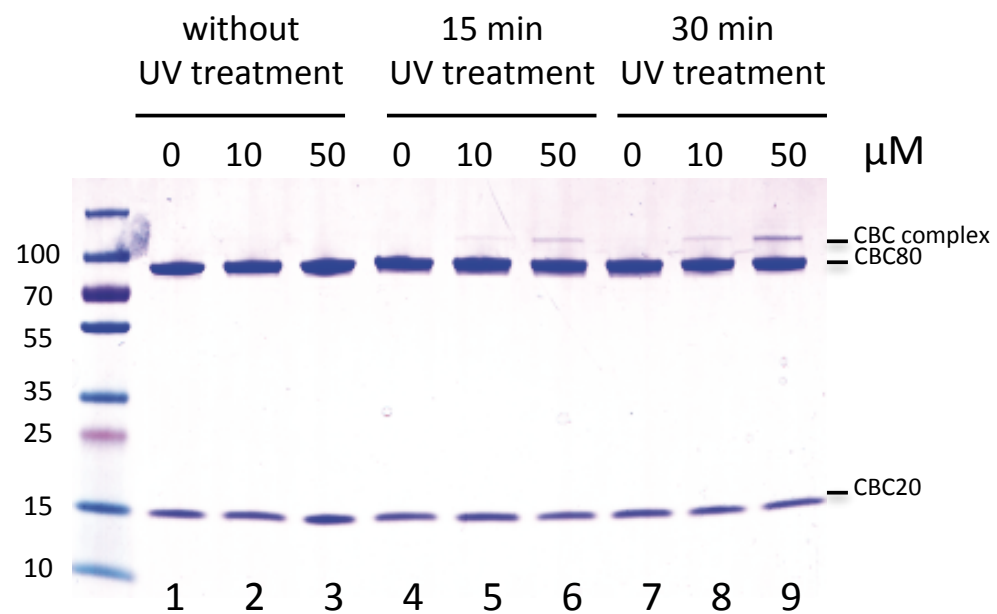**b**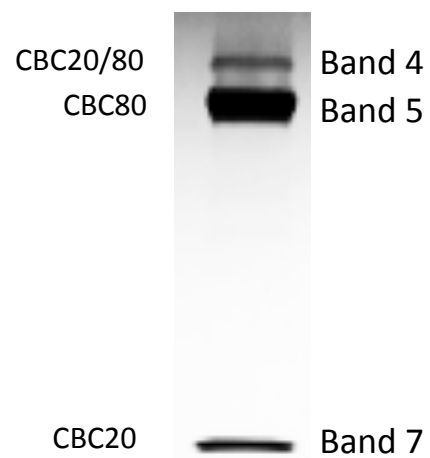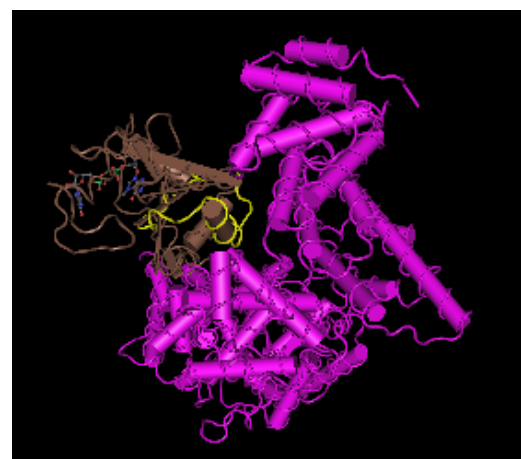

Supplement: Additional file 5: Figure S4. — ABX464 interaction with CBC complex. a. Purified recombinant CBC20 and CBC80 proteins were incubated with the indicated concentrations of ABX464-N-glucuronide and they were either untreated (lanes 1–3), treated during 15 min (lanes 4–6) or 30 min (lanes 7–9) with UV light. Proteins were analyzed by SDS-PAGE and stained with Coumassie blue. ABX464 and ABX464-N-glucuronide promotes UV crosslinking of CBC20 and CBC80. b. Recombinant human CBC was incubated with ABX464-N-glucuronide and treated with UV for 15 min. After crosslinking the proteins were resolved in SDS-PAGE and stained with Coomassie blue (left panel). Stained proteins (Band 4, Band 5 and Band 7) were digested with trypsin and analysed by mass spectrometry (details of mass spectrometry analysis of two independent experiments are in Additional file 6: Table S2). Right panel shows the Ribbon representation of the CBC complex (CBC20 in brown and CBC80 in purple) with the position of the cap RNA (in blue/red) on CBC20 and the putative peptide of CBC20 that interacts with ABX464 (yellow). [file 12977_2015_159_MOESM5_ESM.pdf]
