# Supplementary material for: Long lasting control of viral rebound with a new drug ABX464 targeting Rev – mediated viral RNA biogenesis
Source: Retrovirology. 2015 Apr 9;12:30. doi: 10.1186/s12977-015-0159-3 (PMC4422473; doi:10.1186/s12977-015-0159-3)
Supplement: Additional file 6: Table S2. — Mass spectrometry analysis of CBC complex and ABX464 interaction. [file 12977_2015_159_MOESM6_ESM.pdf]

- Description: Name of the protein.
- MW [kDa]: Calculated molecular weight of the protein.
- # Peptides: Number of distinct peptide sequences in the protein group.
- # PSM: Total number of identified peptide sequences (peptide spectrum matches) for the protein, including those redundantly identified.
- Coverage: Percentage of the protein sequence covered by identified peptides.
- Sequence: Sequence of amino acids that compose the peptide.
- Modifications: Static and dynamic modifications identified in the peptide.
- # Missed Cleavages: Number of cleavage sites in a peptide sequence that a cleavage reagent (trypsin) did not cleave.
- Ion Score: Score for an MS/MS match, based on the calculated probability, P, that the observed match between the experimental data and the database sequence is a random event.
- Exp Value (or Expectation Value): Displays the expectation value, which is a measure of the number of matches with scores equal to or better than the score values that are expected to occur only by chance.The smaller the expectation value is, the better the match is considered.
- Charge: Charge state of the peptide.
- MH+ [Da]: Protonated monoisotopic mass of the peptides, in daltons.
- ΔM [ppm]: Difference between the theoretical mass of the peptide and the experimental mass of the precursor ion.

RT [min]: Retention time when the peptide was observed, in minutes.

Description	MW [kDa]
**Nuclear_CBC20**	**18**

Band4, Experiment 1

# Peptides	# PSMs	Coverage
11	21	44.23

Sequence	# PSMs	Modifications	# Missed Cleavages	IonScore	Exp Value	Charge	MH+ [Da]	ΔM [ppm]	m/z [Da]	RT [min]
KTAcGFcFVEYYSR	1	C4(Carbamidomethyl); C7(Carbamidomethyl)	1	68	1.08315E-06	2	1787.78642	-0.90	894.39685	18.79
TAcGFcFVEYYSR	2	C3(Carbamidomethyl); C6(Carbamidomethyl)	0	63	1.57906E-06	2	1659.69341	0.20	830.35034	21.05
SDSYVELSQYR	3		0	62	8.11968E-06	2	1346.62175	-0.42	673.81451	17.31
ADAENAMR	3		0	61	7.43687E-06	2	877.38323	-0.09	439.19525	10.18
TDWDAGFKEGR	1		1	60	1.73892E-05	2	1281.58867	2.15	641.29797	15.31
DEYRQDYDAGR	2		1	53	2.85318E-05	2	1387.58611	-0.89	694.29669	11.71
TDWDAGFK	2		0	39	0.001203154	2	939.42082	0.11	470.21405	16.87
KIIMGLDK	1		1	35	0.004173594	2	917.54949	0.63	459.27838	13.60
KIImGLDK	2	M4(Oxidation)	1	30	0.019766058	2	933.54363	-0.21	467.27545	11.89
IImGLDK	2	M3(Oxidation)	0	28	0.046420127	2	805.44896	0.13	403.22812	13.67
SGGQVRDEYR	1		1	27	0.031109047	2	1166.55229	-2.25	583.77979	9.68
QDYDAGR	1		0	26	0.017613639	2	824.35259	-0.93	412.67993	9.41

Band7, Experiment 1

# Peptides	# PSMs	Coverage
16	93	72.44

Sequence	# PSMs	Modifications	# Missed Cleavages	IonScore	Exp Value	Charge	MH+ [Da]	ΔM [ppm]	m/z [Da]	RT [min]
KScTLYVGNLSFYTTEEQIYELFSK	1	C3(Carbamidomethyl)	1	99	1.54198E-09	3	3020.46164	0.60	1007.49207	27.76
KTAcGFcFVEYYSR	2	C4(Carbamidomethyl); C7(Carbamidomethyl)	1	89	8.42034E-09	2	1787.78130	-3.77	894.39429	19.81
TAcGFcFVEYYSR	3	C3(Carbamidomethyl); C6(Carbamidomethyl)	0	79	3.47855E-08	2	1659.69231	-0.46	830.34979	21.81
SDSYVELSQYR	24		0	69	1.62009E-06	2	1346.62163	-0.51	673.81445	17.29
ADAENAMR	10		0	61	7.35174E-06	2	877.38323	-0.09	439.19525	11.00
TDWDAGFKEGR	1		1	61	1.21528E-05	2	1281.58757	1.30	641.29742	16.03
QDYDAGR	7		0	52	3.27195E-05	2	824.35399	0.78	412.68063	10.69
TDWDAGFK	9		0	48	0.000148704	2	939.42095	0.24	470.21411	18.69
KIIMGLDK	3		1	43	0.000618743	2	917.54827	-0.70	459.27777	15.57
DEYRQDYDAGR	9		1	43	0.000206435	2	1387.58721	-0.10	694.29724	12.17
DQHFRGDNEEQEK	1		1	41	0.000497233	2	1631.70378	-0.45	816.35553	10.07
SGGQVRDEYR	6		1	36	0.004989665	2	1166.55474	-0.16	583.78101	10.48
KIImGLDK	5	M4(Oxidation)	1	35	0.006917036	2	933.54381	-0.02	467.27554	14.20
IImGLDK	8	M3(Oxidation)	0	33	0.009860969	2	805.44805	-1.01	403.22766	15.67
ALRSDSYVELSQYR	1		1	33	0.011890944	3	1686.84336	-0.75	562.95264	17.47
GDNEEQEKLLK	1		1	31	0.020408342	2	1302.65264	-0.78	651.82996	12.79
ADAENAmR	1	M7(Oxidation)	0	26	0.019329994	2	893.37798	-0.27	447.19263	10.01
ScTLYVGNLSFYTTEEQIYELFSK	1	C2(Carbamidomethyl)	0	23	0.053111169	3	2892.36417	-0.24	964.79291	29.73

Band4, Experiment 2

# Peptides	# PSMs	Coverage
14	29	67.31

Sequence	# PSMs	Modifications	# Missed Cleavages	IonScore	Exp Value	Charge	MH+ [Da]	ΔM [ppm]	m/z [Da]	RT [min]
KTAcGFcFVEYYSR	3	C4(Carbamidomethyl); C7(Carbamidomethyl)	1	102	4.90333E-10	2	1787.78740	-0.36	894.39734	18.40
KScTLYVGNLSFYTTEEQIYELFSK	1	C3(Carbamidomethyl)	1	81	8.34353E-08	3	3020.45780	-0.68	1007.49078	26.50
ScTLYVGNLSFYTTEEQIYELFSK	2	C2(Carbamidomethyl)	0	80	1.31534E-07	2	2892.36577	0.31	1446.68652	28.69
TAcGFcFVEYYSR	2	C3(Carbamidomethyl); C6(Carbamidomethyl)	0	79	3.87754E-08	2	1659.69255	-0.31	830.34991	21.08
GDNEEQEKLLK	2		1	63	1.26881E-05	2	1302.65410	0.34	651.83069	11.71
ADAENAMR	3		0	61	7.76945E-06	2	877.38323	-0.09	439.19525	9.98
SDSYVELSQYR	3		0	60	1.25585E-05	2	1346.62114	-0.87	673.81421	17.32
TDWDAGFK	5		0	48	0.000171918	2	939.42101	0.30	470.21414	17.06
QDYDAGR	1		0	37	0.001370405	2	824.35307	-0.34	412.68018	8.82
KIIMGLDK	2		1	35	0.003651827	2	917.54924	0.36	459.27826	13.78
DEYRQDYDAGR	2		1	31	0.004408896	3	1387.58649	-0.61	463.20035	11.13
IImGLDK	1	M3(Oxidation)	0	28	0.041851046	2	805.44866	-0.25	403.22797	13.04
SGGQVRDEYR	1		1	27	0.034919771	2	1166.55474	-0.16	583.78101	9.06
ALRSDSYVELSQYR	1		1	26	0.054633182	3	1686.84372	-0.53	562.95276	16.20

Band7, Experiment 2

# Peptides	# PSMs	Coverage
15	124	68.59

Sequence	# PSMs	Modifications	# Missed Cleavages	IonScore	Exp Value	Charge	MH+ [Da]	ΔM [ppm]	m/z [Da]	RT [min]
TAcGFcFVEYYSR	4	C3(Carbamidomethyl); C6(Carbamidomethyl)	0	97	5.40005E-10	2	1659.69170	-0.83	830.34949	21.00
KScTLYVGNLSFYTTEEQIYELFSK	1	C3(Carbamidomethyl)	1	83	5.94734E-08	3	3020.46219	0.78	1007.49225	26.44
KTAcGFcFVEYYSR	1	C4(Carbamidomethyl); C7(Carbamidomethyl)	1	74	2.21988E-07	2	1787.78691	-0.63	894.39709	18.43
GDNEEQEKLLK	2		1	72	1.67602E-06	2	1302.65349	-0.12	651.83038	11.66
SDSYVELSQYR	46		0	63	6.69174E-06	2	1346.62163	-0.51	673.81445	20.41
ADAENAMR	16		0	61	7.45401E-06	2	877.38365	0.40	439.19547	10.19
DEYRQDYDAGR	12		1	54	1.7131E-05	2	1387.58782	0.34	694.29755	11.18
ScTLYVGNLSFYTTEEQIYELFSK	1	C2(Carbamidomethyl)	0	50	0.000108073	3	2892.36655	0.58	964.79370	28.64
QDYDAGR	7		0	49	8.50842E-05	2	824.35283	-0.63	412.68005	8.44
TDWDAGFK	13		0	48	0.000158972	2	939.42040	-0.35	470.21384	19.79
KIIMGLDK	2		1	44	0.000521807	2	917.54912	0.23	459.27820	13.46
SGGQVRDEYR	6		1	43	0.000851278	2	1166.55474	-0.16	583.78101	9.32
DQHFRGDNEEQEK	3		1	43	0.000336946	2	1631.70378	-0.45	816.35553	8.81
GDNEEQEK	1		0	36	0.000237817	2	948.39092	0.40	474.69910	6.04
ADAENAmR	2	M7(Oxidation)	0	35	0.002356306	2	893.37700	-1.36	447.19214	9.02
KIImGLDK	4	M4(Oxidation)	1	31	0.014822452	2	933.54332	-0.54	467.27530	12.23
IImGLDK	3	M3(Oxidation)	0	31	0.014994072	2	805.44853	-0.40	403.22791	12.97

Band5, Experiment 2

# Peptides	# PSMs	Coverage
2	2	12.18

Sequence	# PSMs	Modifications	# Missed Cleavages	IonScore	Exp Value	Charge	MH+ [Da]	ΔM [ppm]	m/z [Da]	RT [min]
SDSYVELSQYR	1		0	56	5.17555E-05	2	1346.62212	-0.14	673.81470	16.71
ADAENAMR	1		0	49	0.000226442	2	877.38194	-1.55	439.19461	9.55

Band4, Experiment 2-2

# Peptides	# PSMs	Coverage
14	33	72.44

Sequence	# PSMs	Modifications	# Missed Cleavages	IonScore	Exp Value	Charge	MH+ [Da]	ΔM [ppm]	m/z [Da]	RT [min]
KTAcGFcFVEYYSR	2	C4(Carbamidomethyl); C7(Carbamidomethyl)	1	86	2.55064E-08	2	1787.79057	1.42	894.39893	18.82
TAcGFcFVEYYSR	2	C3(Carbamidomethyl); C6(Carbamidomethyl)	0	74	1.03848E-07	2	1659.69267	-0.24	830.34998	21.02
ADAENAMR	3		0	61	7.26758E-06	2	877.38384	0.61	439.19556	9.70
SDSYVELSQYR	7		0	60	1.39691E-05	2	1346.62297	0.49	673.81512	19.89
TDWDAGFKEGR	1		1	57	3.38867E-05	2	1281.58660	0.53	641.29694	15.17
SGGQVRDEYR	1		1	52	0.000109282	2	1166.55449	-0.37	583.78088	9.24
KScTLYVGNLSFYTTEEQIYELFSK	1	C3(Carbamidomethyl)	1	52	7.08621E-05	3	3020.45926	-0.19	1007.49127	26.86
DQHFRGDNEEQEK	1		1	52	4.69092E-05	2	1631.70867	2.54	816.35797	8.88
TDWDAGFK	7		0	50	0.000104344	2	939.41997	-0.80	470.21362	16.53
GDNEEQEKLLK	2		1	45	0.000809616	3	1302.65348	-0.13	434.88934	11.92
DEYRQDYDAGR	3		1	40	0.000667313	2	1387.58672	-0.45	694.29700	11.17
QDYDAGR	1		0	38	0.00099506	2	824.35350	0.18	412.68039	8.98
KIImGLDK	1	M4(Oxidation)	1	32	0.013865006	2	933.54393	0.11	467.27560	11.77
ALRSDSYVELSQYR	1		1	28	0.037233584	3	1686.84519	0.34	562.95325	16.38

Band5, Experiment 2-2

# Peptides	# PSMs	Coverage
2	2	13.46

Sequence	# PSMs	Modifications	# Missed Cleavages	IonScore	Exp Value	Charge	MH+ [Da]	ΔM [ppm]	m/z [Da]	RT [min]
TDWDAGFK	1		0	28	0.030757892	2	939.41954	-1.26	470.21341	16.79
TAcGFcFVEYYSR	1	C3(Carbamidomethyl); C6(Carbamidomethyl)	0	27	0.035641549	2	1659.69145	-0.97	830.34937	21.02

Band7, Experiment 2-2

# Peptides	# PSMs	Coverage
14	172	68.59

Sequence	# PSMs	Modifications	# Missed Cleavages	IonScore	Exp Value	Charge	MH+ [Da]	ΔM [ppm]	m/z [Da]	RT [min]
KScTLYVGNLSFYTTEEQIYELFSK	1	C3(Carbamidomethyl)	1	102	7.11912E-10	3	3020.45798	-0.62	1007.49084	26.71
ScTLYVGNLSFYTTEEQIYELFSK	4	C2(Carbamidomethyl)	0	99	1.4503E-09	3	2892.36472	-0.05	964.79309	28.85
TAcGFcFVEYYSR	15	C3(Carbamidomethyl); C6(Carbamidomethyl)	0	97	5.22875E-10	2	1659.69206	-0.61	830.34967	22.17
KTAcGFcFVEYYSR	2	C4(Carbamidomethyl); C7(Carbamidomethyl)	1	81	5.5143E-08	2	1787.78728	-0.43	894.39728	18.74
ADAENAMR	27		0	75	2.67541E-07	2	877.38310	-0.22	439.19519	14.42
GDNEEQEKLLK	1		1	72	1.45404E-06	2	1302.65032	-2.56	651.82880	11.91
SDSYVELSQYR	46		0	68	2.20584E-06	2	1346.62078	-1.14	673.81403	16.45
DEYRQDYDAGR	13		1	47	7.05966E-05	2	1387.58696	-0.27	694.29712	11.34
TDWDAGFK	28		0	47	0.000210534	2	939.42070	-0.02	470.21399	23.23
QDYDAGR	10		0	47	0.000149916	2	824.35320	-0.19	412.68024	11.49
SGGQVRDEYR	9		1	46	0.000501971	2	1166.55498	0.05	583.78113	9.54
KIIMGLDK	2		1	42	0.000752506	2	917.55058	1.82	459.27893	13.78
KIImGLDK	4	M4(Oxidation)	1	36	0.005546488	2	933.54314	-0.74	467.27521	12.07
IImGLDK	7	M3(Oxidation)	0	32	0.012806736	2	805.44951	0.81	403.22839	15.13
ADAENAmR	2	M7(Oxidation)	0	27	0.014132914	2	893.37786	-0.40	447.19257	8.84
DQHFRGDNEEQEK	1		1	25	0.020334865	2	1631.70610	0.97	816.35669	8.89

Description	MW [kDa]
**Nuclear_CBC80**	**89.5**

Band4, Experiment 1

# Peptides	# PSMs	Coverage
32	129	48.25

Sequence	# PSMs	Modifications	# Missed Cleavage	IonScore	Exp Value	Charge	MH+ [Da]	ΔM [ppm]	m/z [Da]	RT [min]
TSDANETEDHLESLIcK	2	C16(Carbamidomethyl)	0	103	5.27622E-10	2	1961.87468	-0.55	981.44098	18.45
TLAESDEGKLHVLR	1		1	99	2.51805E-09	2	1567.84209	-1.17	784.42468	14.00
LDTMNTTcVDR	2	C8(Carbamidomethyl)	0	88	1.151E-08	2	1325.58147	-0.76	663.29437	14.05
KTcAAQLVSYPGK	4	C3(Carbamidomethyl)	1	87	4.76786E-08	2	1422.73979	-0.86	711.87354	14.11
LTIYTTLVGLLNAR	5		0	86	7.9076E-09	2	1547.91301	-1.68	774.46014	27.10
MFDYTDDPEGPVMPGSHSVER	4		0	80	2.43523E-08	2	2366.00591	-0.25	1183.50659	18.33
TcAAQLVSYPGK	3	C2(Carbamidomethyl)	0	80	2.8257E-07	2	1294.64543	-0.47	647.82635	16.08
NYNFGGEFVEAMIR	3		0	77	3.06809E-07	2	1646.76225	-0.59	823.88477	24.23
VESAQSEQKNLFLVIFQR	1		1	76	2.36489E-07	3	2136.14206	-1.31	712.71887	23.03
DVPNPNQDDDDDEGFSFNPLK	6		0	74	9.6377E-08	2	2378.00615	0.22	1189.50671	22.68
LDTmNTTcVDR	4	M4(Oxidation); C8(Carbamidomethyl)	0	74	2.00911E-07	2	1341.57659	-0.60	671.29193	12.30
ANNYNEAVYLVR	3		0	74	1.03271E-06	2	1425.71123	-0.64	713.35925	17.93
mFDYTDDPEGPVMPGSHSVER	13	M1(Oxidation)	0	72	1.62781E-07	2	2382.00395	1.07	1191.50562	17.82
cETDGTSVLTPWYK	1	C1(Carbamidomethyl)	0	71	1.01922E-06	2	1656.75542	-1.23	828.88135	20.38
SAcSLESNLEGLAGVLEADLPNYK	1	C3(Carbamidomethyl)	0	69	1.73488E-06	2	2550.23369	-2.21	1275.62048	28.19
WSWEDWSDcLSQDPESPKPK	2	C9(Carbamidomethyl)	0	65	1.02207E-06	3	2477.07163	0.04	826.36206	22.25
FINWFSHHLSNFQFR	7		0	65	5.44751E-06	2	1979.96892	1.26	990.48810	21.38
IEVFVQTLLHLAAK	5		0	63	1.77501E-06	2	1581.93657	0.13	791.47192	24.54
mFDYTDDPEGPVmPGSHSVER	5	M1(Oxidation); M13(Oxidation)	0	63	1.37069E-06	2	2397.99639	0.03	1199.50183	15.96
LFVWEILHSTIR	5		0	59	1.45284E-05	2	1513.85234	-0.18	757.42981	24.53
NYNFGGEFVEAmIR	2	M12(Oxidation)	0	57	1.79396E-05	2	1662.75578	-1.42	831.88153	20.60
ATNDEIFSILK	3		0	56	6.68961E-05	2	1250.66216	-0.48	625.83472	22.14
SKATNDEIFSILK	2		1	56	4.77829E-05	2	1465.79045	0.48	733.39886	19.21
KDGVLEEQIER	6		1	54	0.000106695	2	1315.68511	-0.14	658.34619	14.06
NHPQMIAVLVDK	3		0	53	0.000100693	3	1364.73539	-0.12	455.58331	16.75
SFSHSFSALAK	2		0	51	0.000167937	2	1181.59734	1.99	591.30231	15.18
FImILTEHLVR	4	M3(Oxidation)	0	49	0.000210638	2	1387.77641	-0.20	694.39185	19.89
DGVLEEQIER	1		0	48	0.000423687	2	1187.59014	-0.16	594.29871	16.63
LLPEKLTIYTTLVGLLNAR	1		1	48	4.92219E-05	3	2128.27292	-0.55	710.09583	26.74
LQPGSLPQVLAQATEMLYMR	2		0	46	0.000444627	3	2246.16812	0.38	749.39423	28.86
NHPQmIAVLVDK	4	M5(Oxidation)	0	44	0.000809558	2	1380.72942	-0.76	690.86835	14.29
VmFEVWR	5	M2(Oxidation)	0	40	0.002017993	2	982.48137	-0.19	491.74432	19.47
IFANTESYLK	3		0	40	0.002572047	2	1185.61540	0.30	593.31134	17.46
FIMILTEHLVR	3		0	40	0.0013261	2	1371.78032	-1.06	686.39380	20.78
TLAESDEGK	1		0	37	0.005211683	2	949.44731	-0.02	475.22729	9.81
FVIEENLHcIIK	4	C9(Carbamidomethyl)	0	36	0.006549547	2	1514.80229	-0.88	757.90479	19.17
NLFLVIFQR	3		0	35	0.003090353	2	1149.67742	-0.46	575.34235	25.28
VMFEVWR	2		0	30	0.025520057	2	966.48662	-0.02	483.74695	20.62
LSYHQR	1		0	29	0.03252714	2	803.41545	-0.52	402.21136	9.45

Band5, Experiment 1

# Peptides	# PSMs	Coverage
52	483	74.06

Sequence	# PSMs	Modifications	# Missed Cleavage	IonScore	Exp Value	Charge	MH+ [Da]	ΔM [ppm]	m/z [Da]	RT [min]
SAcSLESNLEGLAGVLEADLPNYK	3	C3(Carbamidomethyl)	0	128	2.64435E-12	2	2550.24150	0.85	1275.62439	28.88
LTIYTTLVGLLNAR	20		0	113	9.44755E-11	2	1547.91435	-0.81	774.46082	28.98
TSDANETEDHLESLIcK	5	C16(Carbamidomethyl)	0	111	1.31923E-10	2	1961.87602	0.14	981.44165	19.29
TQIVDcAAVANWIFSSELSR	4	C6(Carbamidomethyl)	0	109	2.20571E-10	2	2267.11064	-0.86	1134.05896	26.72
WSWEDWSDcLSQDPESPKPK	4	C9(Carbamidomethyl)	0	104	6.44643E-10	3	2477.06705	-1.81	826.36053	22.77
VESAQSEQKNLFLVIFQR	2		1	102	1.10744E-09	2	2136.14336	-0.71	1068.57532	23.83
MFDYTDDPEGPVMPGSHSVER	11		0	98	2.74991E-09	2	2366.00591	-0.25	1183.50659	19.21
KTcAAQLVSYPGK	6	C3(Carbamidomethyl)	1	97	5.13024E-09	2	1422.74016	-0.60	711.87372	14.92
LQPGSLPQVLAQATEMLYMR	7		0	94	7.53909E-09	3	2246.16574	-0.68	749.39343	29.50
ESLKANNYNEAVYLVR	2		1	90	1.69949E-08	2	1882.96367	-1.14	941.98547	17.94
TLAESDEGKLHVLR	5		1	89	2.54567E-08	2	1567.84099	-1.87	784.42413	15.31
LDTMNTTcVDR	2	C8(Carbamidomethyl)	0	88	2.98758E-08	2	1325.58232	-0.11	663.29480	14.36
LDTmNTTcVDR	7	M4(Oxidation); C8(Carbamidomethyl)	0	87	3.30612E-08	2	1341.57695	-0.33	671.29211	13.27
FHEVFKTLAESDEGK	2		1	86	4.53384E-08	2	1736.84819	-0.51	868.92773	16.52
ANNYNEAVYLVR	8		0	82	1.61185E-07	2	1425.71330	0.82	713.36029	19.90
IEVFVQTLLHLAAK	9		0	82	1.09476E-07	2	1581.93547	-0.56	791.47137	25.53
KDGVLEEQIER	9		1	82	2.02836E-07	2	1315.68498	-0.23	658.34613	14.89
FINWFSHHLSNFQFR	32		0	81	1.36106E-07	2	1979.96794	0.77	990.48761	22.75
NYNFGGEFVEAMIR	14		0	81	1.27444E-07	2	1646.76262	-0.37	823.88495	28.47
DAEMDRIFANTESYLK	1		1	79	2.1297E-07	2	1902.89287	1.36	951.95007	22.98
mFDYTDDPEGPVMPGSHSVER	22	M1(Oxidation)	0	76	4.04873E-07	3	2382.00107	-0.14	794.67188	24.36
TcAAQLVSYPGK	5	C2(Carbamidomethyl)	0	74	1.08609E-06	2	1294.64507	-0.76	647.82617	17.56
DVPNPNQDDDDDEGFSFNPLK	10		0	74	6.58141E-07	2	2378.00591	0.12	1189.50659	22.94
SAcSLESNLEGLAGVLEADLPNYKSK	1	C3(Carbamidomethyl)	1	73	8.53727E-07	3	2765.35727	-3.27	922.45728	26.96
SKATNDEIFSILK	3		1	73	9.97282E-07	2	1465.78948	-0.19	733.39838	20.06
LFVWEILHSTIR	49		0	72	9.62319E-07	2	1513.85222	-0.26	757.42975	25.01
LLPEKLTIYTTLVGLLNAR	5		1	70	1.61183E-06	3	2128.27292	-0.55	710.09583	28.04
NYNFGGEFVEAmIR	11	M12(Oxidation)	0	70	1.69168E-06	2	1662.75725	-0.54	831.88226	21.91
cETDGTSVLTPWYK	4	C1(Carbamidomethyl)	0	70	1.85063E-06	2	1656.75640	-0.64	828.88184	21.25
mFDYTDDPEGPVmPGSHSVER	12	M1(Oxidation); M13(Oxidation)	0	67	3.15732E-06	2	2397.99761	0.54	1199.50244	16.77
TcAAQLVSYPGKNK	1	C2(Carbamidomethyl)	1	65	9.45698E-06	2	1536.78264	-0.85	768.89496	14.57
SFSHSFSALAK	6		0	64	9.69285E-06	2	1181.59551	0.44	591.30139	16.33
NHPQMIAVLVDK	8		0	63	9.08173E-06	3	1364.73520	-0.26	455.58325	18.23
FIMILTEHLVR	19		0	60	1.69949E-05	2	1371.78118	-0.43	686.39423	21.90
LQEKVESAQSEQK	2		1	59	3.44903E-05	3	1503.76545	0.30	501.92667	10.37
VIFRMFDYTDDPEGPVMPGSHSVER	2		1	59	2.18937E-05	3	2881.32877	0.09	961.11444	21.75
ATNDEIFSILK	13		0	56	6.65887E-05	2	1250.66252	-0.18	625.83490	23.09
IFANTESYLK	8		0	56	6.59638E-05	2	1185.61492	-0.11	593.31110	18.55
FImILTEHLVR	15	M3(Oxidation)	0	55	5.27617E-05	2	1387.77739	0.51	694.39233	23.50
NHPQMIAVLVDKMIR	2		1	54	7.1667E-05	3	1764.96036	-0.50	588.99164	22.17
VmFEVWR	21	M2(Oxidation)	0	52	0.000120481	2	982.48149	-0.06	491.74438	20.96
DGVLEEQIER	4		0	52	0.000186657	2	1187.59062	0.25	594.29895	17.41
FVIEENLHcIIK	9	C9(Carbamidomethyl)	0	51	0.000192846	2	1514.80242	-0.80	757.90485	19.97
NHPQmIAVLVDK	10	M5(Oxidation)	0	49	0.000244971	3	1380.73011	-0.26	460.91489	15.21
THVPMLQVWTADKPHPQEEYLDcLW	5	C23(Carbamidomethyl)	0	49	0.00021297	4	3661.78969	-0.95	916.20288	24.39
IQKELEEAK	1		1	48	0.000679835	2	1087.59990	0.44	544.30359	10.95
FLSDLVNcHVIAAPSMVAMFENFVSV	1	C8(Carbamidomethyl)	0	45	0.000573217	4	4078.95693	-3.53	1020.49469	30.72
ELEEAKEK	1		1	41	0.002662417	2	975.49907	-0.31	488.25317	9.11
LQPGSLPQVLAQATEmLYmR	1	M16(Oxidation); M19(Oxidation)	0	40	0.001854894	3	2278.15628	-0.36	760.05695	27.12
RDWYVYAFLSSLPWVGK	3		1	39	0.001978422	3	2087.07199	-1.43	696.36218	29.53
TLAESDEGK	2		0	39	0.002985231	2	949.44737	0.05	475.22733	10.15
KDAEMDR	1		1	39	0.00203385	2	864.38762	-0.50	432.69745	7.66
NLFLVIFQR	22		0	38	0.002879513	2	1149.67729	-0.56	575.34229	28.56
LQPGSLPQVLAQATEmLYMR	22	M16(Oxidation)	0	37	0.003430197	2	2262.16338	0.52	1131.58533	27.61
HILRPYLAFDSILcEALQHNLPPFTPPP	6	C14(Carbamidomethyl)	0	36	0.004659282	6	4569.28869	0.85	762.38751	27.47
THVPmLQVWTADKPHPQEEYLDcLW	3	M5(Oxidation); C23(Carbamidomethyl)	0	35	0.005097052	4	3677.78921	0.31	920.20276	23.44
VMFEVWR	13		0	33	0.013895739	2	966.48631	-0.34	483.74680	22.45
DWYVYAFLSSLPWVGK	1		0	32	0.014265351	2	1930.97429	0.22	965.99078	32.57
LSYHQR	1		0	31	0.023401617	2	803.41570	-0.22	402.21149	9.77
LLcTVAR	1	C3(Carbamidomethyl)	0	28	0.050745417	2	832.47038	-0.71	416.73883	13.60
LFVWEILHSTIRK	1		1	26	0.038398921	3	1641.94736	-0.14	547.98730	23.07
SHWKER	1		1	26	0.039022854	2	842.42632	-0.56	421.71680	9.02
HILRPYLAFDSILcEALQHNLPPFTPPP	2	C14(Carbamidomethyl); M37(Oxidation)	0	20	0.153221258	4	4585.29409	3.14	1147.07898	26.83

Band4, Experiment 2

# Peptides	# PSMs	Coverage
44	199	67.32

Sequence	# PSMs	Modifications	# Missed Cleavage	IonScore	Exp Value	Charge	MH+ [Da]	ΔM [ppm]	m/z [Da]	RT [min]
SAcSLESNLEGLAGVLEADLPNYK	3	C3(Carbamidomethyl)	0	130	1.36033E-12	2	2550.24346	1.62	1275.62537	27.74
TQIVDcAAVANWIFSSELSR	4	C6(Carbamidomethyl)	0	122	1.10864E-11	2	2267.11357	0.43	1134.06042	26.22
LQPGSLPQVLAQATEMLYMR	6		0	111	1.15661E-10	3	2246.16611	-0.52	749.39355	28.51
TSDANETEDHLESLIcK	7	C16(Carbamidomethyl)	0	104	4.27881E-10	2	1961.87395	-0.92	981.44061	18.41
mFDYTDDPEGPVMPGSHSVER	9	M1(Oxidation)	0	103	1.41109E-10	2	2381.99834	-1.29	1191.50281	17.05
KTcAAQLVSYPGK	5	C3(Carbamidomethyl)	1	101	1.84135E-09	2	1422.74040	-0.43	711.87384	13.79
MFDYTDDPEGPVMPGSHSVER	9		0	100	3.09426E-10	2	2366.00444	-0.87	1183.50586	18.36
WSWEDWSDcLSQDPESPKPK	11	C9(Carbamidomethyl)	0	92	1.98371E-09	2	2477.07134	-0.08	1239.03931	22.23
TLAESDEGKLHVLR	3		1	89	2.55271E-08	2	1567.84477	0.54	784.42603	13.69
LDTMNTTcVDR	3	C8(Carbamidomethyl)	0	87	1.22984E-08	2	1325.58196	-0.39	663.29462	13.78
LTIYTTLVGLLNAR	4		0	84	1.26054E-08	3	1547.91501	-0.39	516.64319	26.82
cETDGTSVLTPWYK	4	C1(Carbamidomethyl)	0	83	6.5249E-08	2	1656.75835	0.54	828.88281	20.06
ANNYNEAVYLVR	8		0	81	1.87703E-07	2	1425.71269	0.39	713.35999	19.52
SKATNDEIFSILK	2		1	80	1.72456E-07	2	1465.78838	-0.94	733.39783	18.91
TcAAQLVSYPGK	5	C2(Carbamidomethyl)	0	80	2.75193E-07	2	1294.64617	0.09	647.82672	15.74
DVPNPNQDDDDDEGFSFNPLK	8		0	76	8.00859E-08	3	2378.00406	-0.66	793.33954	21.96
IEVFVQTLLHLAAK	5		0	76	9.51046E-08	2	1581.93511	-0.79	791.47119	23.92
LDTmNTTcVDR	4	M4(Oxidation); C8(Carbamidomethyl)	0	75	1.65123E-07	2	1341.57707	-0.24	671.29218	11.95
VESAQSEQKNLFLVIFQR	2		1	72	5.16436E-07	2	2136.14580	0.43	1068.57654	22.75
NYNFGGEFVEAMIR	5		0	71	1.0396E-06	2	1646.76213	-0.67	823.88470	23.58
KDGVLEEQIER	5		1	69	3.88281E-06	2	1315.68498	-0.23	658.34613	13.46
NHPQMIAVLVDK	6		0	63	9.35769E-06	3	1364.73493	-0.46	455.58316	16.53
FLSDLVNcHVIAAPSMVAMFENFVSV	2	C8(Carbamidomethyl)	0	59	7.9893E-06	3	4078.95920	-2.97	1360.32458	29.56
LQEKVESAQSEQK	2		1	59	2.894E-05	2	1503.76372	-0.85	752.38550	9.51
SFSHSFSALAK	5		0	58	2.86365E-05	2	1181.59184	-2.66	591.29956	14.53
FINWFSHHLSNFQFR	1		0	57	4.3064E-05	2	1979.97002	1.82	990.48865	20.95
ESLKANNYNEAVYLVR	2		1	56	4.41445E-05	3	1882.96689	0.57	628.32715	16.91
ATNDEIFSILK	10		0	56	6.40325E-05	2	1250.66252	-0.18	625.83490	21.87
IFANTESYLK	3		0	56	6.74961E-05	2	1185.61553	0.40	593.31140	17.54
VmFEVWR	2	M2(Oxidation)	0	52	0.000121876	2	982.48125	-0.31	491.74426	18.59
LFVWEILHSTIR	5		0	52	7.03595E-05	2	1513.85185	-0.51	757.42957	23.89
TcAAQLVSYPGKNK	1	C2(Carbamidomethyl)	1	50	0.000288804	2	1536.78411	0.10	768.89569	13.62
FImILTEHLVR	3	M3(Oxidation)	0	49	0.000214156	2	1387.77580	-0.64	694.39154	19.35
mFDYTDDPEGPVmPGSHSVER	1	M1(Oxidation); M13(Oxidation)	0	49	4.44177E-05	3	2397.99643	0.05	800.00366	15.70
DGVLEEQIER	1		0	48	0.000466708	2	1187.59050	0.15	594.29889	16.39
TLAESDEGK	6		0	47	0.000486383	2	949.44756	0.24	475.22742	9.55
DWYVYAFLSSLPWVGK	1		0	46	0.000484243	2	1930.97002	-1.99	965.98865	31.68
FIMILTEHLVR	5		0	45	0.000340697	3	1371.78119	-0.42	457.93192	20.89
FVIEENLHcIIK	5	C9(Carbamidomethyl)	0	44	0.000754861	2	1514.80144	-1.44	757.90436	18.91
NHPQmIAVLVDK	2	M5(Oxidation)	0	44	0.00086719	3	1380.73056	0.07	460.91504	14.05
NYNFGGEFVEAmIR	1	M12(Oxidation)	0	43	0.000433323	2	1662.75591	-1.35	831.88159	20.29
LQPGSLPQVLAQATEMLYmR	5	M19(Oxidation)	0	39	0.001634416	3	2262.15635	-2.59	754.72363	27.64
MNKHVLK	1		1	35	0.003658978	2	869.50206	-0.63	435.25467	7.10
LQPGSLPQVLAQATEmLYmR	1	M16(Oxidation); M19(Oxidation)	0	35	0.005294511	3	2278.15592	-0.52	760.05682	25.84
NLFLVIFQR	4		0	35	0.002906825	2	1149.67827	0.29	575.34277	24.87
IQKELEEAK	1		1	34	0.012344785	2	1087.59953	0.10	544.30341	9.92
ELEEAKEK	1		1	34	0.012867286	2	975.49925	-0.12	488.25327	7.50
LSYHQR	3		0	32	0.017508376	2	803.41576	-0.14	402.21152	8.92
LLcTVAR	2	C3(Carbamidomethyl)	0	31	0.029077592	2	832.47051	-0.56	416.73889	13.01
VMFEVWR	2		0	30	0.02635613	2	966.48638	-0.27	483.74683	20.06
QLKESLK	1		1	30	0.026897489	2	845.50878	-0.42	423.25803	9.53
LLPEKLTIYTTLVGLLNAR	1		1	28	0.00453478	3	2128.27530	0.57	710.09662	26.33
IPLNYHIVEVIFAELFQLPAPPHIDVMY	1	C36(Carbamidomethyl)	0	19	0.036690718	5	4350.33591	0.84	870.87300	33.17

Band5, Experiment 2

# Peptides	# PSMs	Coverage
65	823	85.08

Sequence	# PSMs	Modifications	# Missed Cleavage	IonScore	Exp Value	Charge	MH+ [Da]	ΔM [ppm]	m/z [Da]	RT [min]
SAcSLESNLEGLAGVLEADLPNYK	27	C3(Carbamidomethyl)	0	142	1.35194E-13	2	2550.23931	-0.01	1275.62329	28.71
TQIVDcAAVANWIFSSELSR	14	C6(Carbamidomethyl)	0	123	1.09133E-11	2	2267.10893	-1.61	1134.05811	26.96
TSDANETEDHLESLIcK	14	C16(Carbamidomethyl)	0	113	1.09133E-10	2	1961.88225	3.31	981.44476	16.22
mFDYTDDPEGPVMPGSHSVER	12	M1(Oxidation)	0	113	1.09385E-10	2	2382.00615	1.99	1191.50671	17.00
MFDYTDDPEGPVMPGSHSVER	16		0	110	2.16749E-10	2	2366.00688	0.17	1183.50708	18.60
VESAQSEQKNLFLVIFQR	2		1	109	2.43757E-10	2	2136.14433	-0.25	1068.57581	22.68
KTcAAQLVSYPGK	12	C3(Carbamidomethyl)	1	107	5.15157E-10	2	1422.74114	0.09	711.87421	13.67
WSWEDWSDcLSQDPESPKPK	31	C9(Carbamidomethyl)	0	105	5.87431E-10	2	2477.07012	-0.57	1239.03870	21.85
SAcSLESNLEGLAGVLEADLPNYKSK	1	C3(Carbamidomethyl)	1	105	5.99731E-10	3	2765.37027	1.43	922.46161	25.92
LTIYTTLVGLLNAR	35		0	100	1.9859E-09	2	1547.91557	-0.02	774.46143	27.11
LQPGSLPQVLAQATEMLYMR	30		0	100	2.07949E-09	3	2246.16721	-0.03	749.39392	31.24
VGEKSAcSLESNLEGLAGVLEADLPNY	1	C7(Carbamidomethyl)	1	97	3.68941E-09	3	2963.46622	-0.19	988.49359	26.15
cETDGTSVLTPWYK	10	C1(Carbamidomethyl)	0	96	5.13992E-09	2	1656.76140	2.38	828.88434	21.47
LLPEKLTIYTTLVGLLNAR	5		1	96	5.59702E-09	3	2128.27256	-0.72	710.09570	26.27
TLAESDEGKLHVLR	6		1	92	1.15069E-08	2	1567.84075	-2.03	784.42401	14.38
ESLKANNYNEAVYLVR	2		1	90	1.85335E-08	2	1882.96526	-0.29	941.98627	16.89
YGDESSNSLPGHSVALcLAVAFKSK	2	C17(Carbamidomethyl)	1	90	1.92733E-08	3	2637.29697	-0.33	879.77051	19.99
NYNFGGEFVEAMIR	33		0	90	1.99047E-08	2	1646.77068	4.52	823.88898	32.32
YGDESSNSLPGHSVALcLAVAFK	10	C17(Carbamidomethyl)	0	89	2.43757E-08	3	2422.17075	-0.04	808.06177	22.47
DVPNPNQDDDDDEGFSFNPLK	13		0	89	2.55245E-08	2	2378.00469	-0.39	1189.50598	22.29
LDTMNTTcVDR	13	C8(Carbamidomethyl)	0	88	3.35704E-08	2	1325.58306	0.44	663.29517	13.59
ANNYNEAVYLVR	36		0	82	1.60814E-07	2	1425.71355	0.99	713.36041	22.31
FHEVFKTLAESDEGK	2		1	82	1.24726E-07	2	1736.84807	-0.58	868.92767	14.73
TcAAQLVSYPGK	13	C2(Carbamidomethyl)	0	80	2.86514E-07	2	1294.64702	0.75	647.82715	16.34
DWYVYAFLSSLPWVGK	3		0	78	3.51896E-07	2	1930.97368	-0.09	965.99048	31.83
NHPQMIAVLVDKMIR	4		1	78	3.54778E-07	3	1764.96054	-0.39	588.99170	20.23
FINWFSHHLSNFQFR	54		0	77	3.66401E-07	2	1979.96269	-1.88	990.48499	21.53
LDTmNTTcVDR	8	M4(Oxidation); C8(Carbamidomethyl)	0	77	4.2752E-07	2	1341.58000	1.95	671.29364	12.01
IEVFVQTLLHLAAK	65		0	77	4.36472E-07	2	1581.93425	-1.33	791.47076	32.14
FLSDLVNcHVIAAPSMVAMFENFVSV	12	C8(Carbamidomethyl)	0	75	6.10881E-07	3	4078.97348	0.53	1360.32935	31.26
SFSHSFSALAKFHEVFK	1		1	74	8.07154E-07	2	1968.99553	-0.61	985.00140	18.34
KDGVLEEQIER	10		1	72	1.8413E-06	2	1315.68559	0.23	658.34644	14.08
SKATNDEIFSILK	5		1	70	2.0939E-06	2	1465.78838	-0.94	733.39783	18.83
DGVLEEQIER	2		0	70	2.85259E-06	2	1187.59441	3.44	594.30084	16.69
NYNFGGEFVEAmIR	3	M12(Oxidation)	0	69	2.44882E-06	2	1662.75493	-1.93	831.88110	23.76
mFDYTDDPEGPVmPGSHSVER	3	M1(Oxidation); M13(Oxidation)	0	66	4.67688E-06	2	2397.99834	0.84	1199.50281	15.62
FLSDLVNcHVIAAPSmVAmFENFVSV	2	C8(Carbamidomethyl); M16(Oxidation); M19(Oxidation)	0	66	5.16365E-06	4	4110.98012	4.61	1028.50049	31.68
NHPQMIAVLVDK	20		0	66	5.48525E-06	2	1364.73430	-0.92	682.87079	17.78
SFSHSFSALAK	8		0	66	6.04577E-06	2	1181.59490	-0.08	591.30109	15.56
LQEKVESAQSEQK	3		1	61	1.83065E-05	2	1503.76433	-0.44	752.38580	9.82
FIMILTEHLVR	43		0	60	1.96769E-05	2	1371.78142	-0.26	686.39435	22.87
LFVWEILHSTIR	45		0	60	2.12303E-05	2	1513.85124	-0.91	757.42926	24.15
ATNDEIFSILK	22		0	56	6.69628E-05	2	1250.66289	0.11	625.83508	24.45
IFANTESYLK	15		0	56	6.66526E-05	2	1185.61577	0.61	593.31152	17.51
LQPGSLPQVLAQATEmLYMR	14	M16(Oxidation)	0	55	6.01114E-05	3	2262.15964	-1.13	754.72473	26.54
FImILTEHLVR	9	M3(Oxidation)	0	55	6.20807E-05	2	1387.77641	-0.20	694.39185	19.96
FLSDLVNcHVIAAPSmVAMFENFVSV	20	C8(Carbamidomethyl); M16(Oxidation)	0	53	9.01481E-05	4	4094.96352	-0.66	1024.49634	27.75
TcAAQLVSYPGKNK	2	C2(Carbamidomethyl)	1	51	0.00019116	2	1536.78508	0.74	768.89618	13.63
VESAQSEQK	1		0	50	0.000262977	2	1005.48454	-0.23	503.24591	6.29
FVIEENLHcIIK	10	C9(Carbamidomethyl)	0	50	0.000267311	2	1514.80303	-0.40	757.90515	19.17
TLAESDEGK	8		0	49	0.000302775	2	949.44890	1.65	475.22809	9.16
THVPmLQVWTADKPHPQEEYLDcLW	5	M5(Oxidation); C23(Carbamidomethyl)	0	49	0.000258795	4	3677.78481	-0.89	920.20166	22.05
NHPQmIAVLVDK	9	M5(Oxidation)	0	49	0.000266756	2	1380.73003	-0.32	690.86865	14.37
THVPMLQVWTADKPHPQEEYLDcLW	5	C23(Carbamidomethyl)	0	48	0.000283111	4	3661.78945	-1.01	916.20282	23.94

IQKELEEAK	2		1	47	0.000625869	2	1087.59917	-0.23	544.30322	9.89
RDWYVYAFLSSLPWVGK	3		1	47	0.000415869	2	2087.07671	0.83	1044.04199	27.52
ILRLLcTVAR	1	C6(Carbamidomethyl)	1	47	0.000438487	3	1214.74060	0.30	405.58505	17.00
KDAEMDR	1		1	45	0.000665207	2	864.38780	-0.29	432.69754	6.45
HILRPYLAFDSILcEALQHNLPPFTPPP	6	C14(Carbamidomethyl)	0	44	0.000803446	4	4569.28139	-0.74	1143.07581	24.80
FLSDLVNcHVIAAPSMVAMFENFVSV	1	C8(Carbamidomethyl)	1	44	0.000882992	4	4235.07192	-0.12	1059.52344	28.15
DVPNPNQDDDDDEGFSFNPLKIEVFV	1		1	41	0.001489212	4	3940.92006	-1.03	985.98547	32.00
VmFEVWR	1	M2(Oxidation)	0	39	0.002553973	2	982.48192	0.37	491.74460	18.56
IPLNYHIVEVIFAELFQLPAPPHIDVMY	4	C36(Carbamidomethyl)	0	39	0.002722429	4	4350.32924	-0.69	1088.33777	33.12
VIFRMFDYTDDPEGPVMPGSHSVER	1		1	37	0.004064027	4	2881.32290	-1.95	721.08618	20.43
ELEEAKEK	2		1	36	0.007022423	2	975.49956	0.19	488.25342	7.49
VMFEVWR	11		0	36	0.007394037	2	966.48656	-0.09	483.74692	21.05
FVREVLEK	2		1	35	0.00592866	2	1019.59184	3.32	510.29956	12.77
NLFLVIFQR	16		0	35	0.006652066	2	1149.67790	-0.03	575.34259	31.43
LFVWEILHSTIRK	1		1	34	0.007412361	3	1641.94699	-0.36	547.98718	21.29
EVLEKcMR	1	C6(Carbamidomethyl)	1	34	0.011386517	2	1064.52336	0.55	532.76532	10.91
FVIEENLHcIIKSHWK	2	C9(Carbamidomethyl)	1	33	0.009203575	2	2053.06767	-0.58	1027.03748	18.49
LSYHQR	7		0	33	0.012648117	2	803.41606	0.24	402.21167	8.18
LQPGSLPQVLAQATEMLYMRLDTMN	1	C28(Carbamidomethyl)	1	31	0.015274133	4	3552.71914	-3.60	888.93524	29.47
LQPGSLPQVLAQATEmLYmR	1	M16(Oxidation); M19(Oxidation)	0	31	0.016980738	2	2278.14995	-3.14	1139.57861	25.78
LLcTVAR	4	C3(Carbamidomethyl)	0	31	0.029550065	2	832.47093	-0.05	416.73911	14.21
MNKHVLK	1		1	29	0.024207869	2	869.50243	-0.21	435.25485	7.29
SHWKER	1		1	27	0.0398985	2	842.42626	-0.64	421.71677	6.84
HILRPYLAFDSILcEALQHNLPPFTPPP	1	C14(Carbamidomethyl); M37(Oxidation)	0	23	0.103028308	4	4585.28335	0.80	1147.07629	24.46

Band7, Experiment 2

# Peptides	# PSMs	Coverage
3	3	3.76

Sequence	# PSMs	Modifications	# Missed Cleavage	IonScore	Exp Value	Charge	MH+ [Da]	ΔM [ppm]	m/z [Da]	RT [min]
LDTMNTTcVDR	1	C8(Carbamidomethyl)	0	59	8.97117E-06	2	1325.58232	-0.11	663.29480	13.50
KDGVLEEQIER	1		1	30	0.032281855	2	1315.68413	-0.88	658.34570	13.54
LLcTVAR	1	C3(Carbamidomethyl)	0	28	0.05291252	2	832.47118	0.24	416.73923	12.70

Band4, Experiment 2-2

# Peptides	# PSMs	Coverage
39	148	53.31

Sequence	# PSMs	Modifications	# Missed Cleavage	IonScore	Exp Value	Charge	MH+ [Da]	ΔM [ppm]	m/z [Da]	RT [min]
TQIVDcAAVANWIFSSELSR	2	C6(Carbamidomethyl)	0	108	2.83059E-10	2	2267.11235	-0.11	1134.05981	26.04
TSDANETEDHLESLIcK	6	C16(Carbamidomethyl)	0	108	1.76692E-10	2	1961.87566	-0.05	981.44147	18.28
KTcAAQLVSYPGK	6	C3(Carbamidomethyl)	1	105	8.62859E-10	2	1422.74089	-0.09	711.87408	14.03
cETDGTSVLTPWYK	7	C1(Carbamidomethyl)	0	102	7.13158E-10	2	1656.74773	-5.87	828.87750	24.52
MFDYTDDPEGPVMPGSHSVER	4		0	99	3.12277E-10	2	2366.00688	0.17	1183.50708	18.21
LDTMNTTcVDR	2	C8(Carbamidomethyl)	0	88	1.11905E-08	2	1325.58220	-0.21	663.29474	13.71
LDTmNTTcVDR	4	M4(Oxidation); C8(Carbamidomethyl)	0	87	9.92675E-09	2	1341.57683	-0.42	671.29205	12.10
WSWEDWSDcLSQDPESPKPK	3	C9(Carbamidomethyl)	0	85	9.30858E-09	2	2477.06621	-2.15	1239.03674	22.16
mFDYTDDPEGPVMPGSHSVER	7	M1(Oxidation)	0	83	1.17269E-08	2	2382.00298	0.66	1191.50513	17.29
NYNFGGEFVEAMIR	3		0	82	8.35318E-08	2	1646.76348	0.15	823.88538	23.88
TcAAQLVSYPGK	8	C2(Carbamidomethyl)	0	80	2.8031E-07	2	1294.64641	0.28	647.82684	15.81
TLAESDEGKLHVLR	3		1	76	3.86837E-07	2	1567.84331	-0.39	784.42529	13.94
DVPNPNQDDDDDEGFSFNPLK	6		0	74	9.9624E-08	2	2378.00713	0.63	1189.50720	21.96
ANNYNEAVYLVR	8		0	74	1.17653E-06	2	1425.71477	1.84	713.36102	20.70
LTIYTTLVGLLNAR	2		0	73	1.57874E-07	2	1547.91545	-0.10	774.46136	27.19
KDGVLEEQIER	4		1	70	3.26097E-06	2	1315.68462	-0.51	658.34595	14.29
mFDYTDDPEGPVmPGSHSVER	3	M1(Oxidation); M13(Oxidation)	0	69	3.01471E-07	2	2397.99845	0.89	1199.50286	15.77
LQEKVESAQSEQK	2		1	68	4.04109E-06	2	1503.76372	-0.85	752.38550	9.63
SAcSLESNLEGLAGVLEADLPNYK	2	C3(Carbamidomethyl)	0	68	2.28509E-06	2	2550.24468	2.10	1275.62598	27.98
LQPGSLPQVLAQATEMLYMR	2		0	66	4.0644E-06	3	2246.16812	0.38	749.39423	28.87
SKATNDEIFSILK	2		1	61	1.60257E-05	2	1465.78911	-0.44	733.39819	19.33
LFVWEILHSTIR	2		0	59	1.15298E-05	2	1513.85344	0.54	757.43036	23.95
NHPQMIAVLVDK	2		0	59	2.33346E-05	2	1364.73540	-0.11	682.87134	16.87
TcAAQLVSYPGKNK	2	C2(Carbamidomethyl)	1	59	3.09502E-05	2	1536.78557	1.06	768.89642	13.86
IFANTESYLK	6		0	56	6.09966E-05	2	1185.61443	-0.53	593.31085	17.40
ATNDEIFSILK	12		0	56	6.7427E-05	2	1250.66289	0.11	625.83508	23.12
NYNFGGEFVEAmIR	1	M12(Oxidation)	0	56	2.6105E-05	2	1662.75615	-1.20	831.88171	20.52
IQKELEEAK	1		1	52	0.000210612	2	1087.59892	-0.46	544.30310	10.07
SFSHSFSALAK	3		0	49	0.000263257	2	1181.59404	-0.80	591.30066	15.14
DGVLEEQIER	2		0	47	0.000505877	2	1187.59026	-0.05	594.29877	16.72
ESLKANNYNEAVYLVR	1		1	44	0.000725645	3	1882.96341	-1.27	628.32599	17.22
NHPQmIAVLVDK	2	M5(Oxidation)	0	43	0.000976242	2	1380.73039	-0.05	690.86884	14.32
EVLEKcMR	1	C6(Carbamidomethyl)	1	42	0.001509855	2	1064.52275	-0.02	532.76501	11.10
TLAESDEGK	5		0	41	0.002137854	2	949.44835	1.07	475.22781	9.34
KDAEMDR	1		1	40	0.001369427	2	864.38793	-0.14	432.69760	6.65
VmFEVWR	1	M2(Oxidation)	0	39	0.002736632	2	982.48161	0.06	491.74445	18.85
ELEEAKEK	2		1	35	0.008759654	2	975.49968	0.32	488.25348	9.56
IEVFVQTLLHLAAK	3		0	35	0.001148687	3	1581.93509	-0.80	527.98322	24.30
LLPEKLTIYTTLVGLLNAR	1		1	35	0.000893631	3	2128.27384	-0.12	710.09613	26.71
LSYHQR	3		0	35	0.009209708	2	803.41570	-0.22	402.21149	7.02
NLFLVIFQR	2		0	34	0.003706876	2	1149.67729	-0.56	575.34229	25.07
FVREVLEK	1		1	33	0.006482172	2	1019.58898	0.50	510.29813	12.96
FIMILTEHLVR	2		0	33	0.007123973	2	1371.78179	0.01	686.39453	20.97
LLcTVAR	1	C3(Carbamidomethyl)	0	32	0.019568541	2	832.47106	0.10	416.73917	13.14
VMFEVWR	3		0	30	0.029034148	2	966.48711	0.48	483.74719	21.06
LQPGSLPQVLAQATEMLYmR	1	M19(Oxidation)	0	26	0.037099025	3	2262.15635	-2.59	754.72363	28.00
FImILTEHLVR	1	M3(Oxidation)	0	26	0.040598907	2	1387.77654	-0.11	694.39191	19.70

Band5, Experiment 2-2

# Peptides	# PSMs	Coverage
71	915	85.99

Sequence	# PSMs	Modifications	# Missed Cleavage	IonScore	Exp Value	Charge	MH+ [Da]	ΔM [ppm]	m/z [Da]	RT [min]
SAcSLESNLEGLAGVLEADLPNYK	28	C3(Carbamidomethyl)	0	182	1.19112E-17	2	2550.23613	-1.25	1275.62170	28.51
TSDANETEDHLESLIcK	14	C16(Carbamidomethyl)	0	126	4.54943E-12	2	1961.87444	-0.67	981.44086	16.40
TQIVDcAAVANWIFSSELSR	23	C6(Carbamidomethyl)	0	124	8.74896E-12	2	2267.11186	-0.32	1134.05957	26.27
WSWEDWSDcLSQDPESPKPK	17	C9(Carbamidomethyl)	0	123	1.06404E-11	2	2477.06523	-2.54	1239.03625	22.05
LDTmNTTcVDR	8	M4(Oxidation); C8(Carbamidomethyl)	0	115	6.76015E-11	2	1341.57756	0.13	671.29242	11.71
VESAQSEQKNLFLVIFQR	2		1	114	7.14425E-11	2	2136.14800	1.46	1068.57764	23.06
LTIYTTLVGLLNAR	63		0	113	1.06159E-10	2	1547.91606	0.29	774.46167	34.00
LQPGSLPQVLAQATEMLYMR	14		0	111	1.41565E-10	3	2246.16684	-0.20	749.39380	28.71
MFDYTDDPEGPVMPGSHSVER	7		0	98	3.08999E-09	2	2366.00810	0.68	1183.50769	18.44
KTcAAQLVSYPGK	12	C3(Carbamidomethyl)	1	98	4.3445E-09	2	1422.74101	0.00	711.87415	13.91
DWYVYAFLSSLPWVGK	6		0	97	3.85754E-09	2	1930.97392	0.03	965.99060	31.96
TLAESDEGKLHVLR	14		1	97	4.05468E-09	2	1567.84221	-1.09	784.42474	14.56
DVPNPNQDDDDDEGFSFNPLK	16		0	96	4.66613E-09	2	2378.00493	-0.29	1189.50610	23.27
LLPEKLTIYTTLVGLLNAR	15		1	96	5.24755E-09	3	2128.27365	-0.21	710.09607	27.25
ESLKANNYNEAVYLVR	2		1	95	6.47078E-09	2	1882.96379	-1.07	941.98553	17.11
VGEKSAcSLESNLEGLAGVLEADLPNY	1	C7(Carbamidomethyl)	1	95	6.59108E-09	3	2963.47190	1.72	988.49548	26.44
YGDESSNSLPGHSVALcLAVAFK	52	C17(Carbamidomethyl)	0	89	2.57606E-08	3	2422.16819	-1.09	808.06091	25.17
LDTMNTTcVDR	6	C8(Carbamidomethyl)	0	87	3.55596E-08	2	1325.58183	-0.48	663.29456	13.34
FINWFSHHLSNFQFR	39		0	85	6.50065E-08	2	1979.96501	-0.71	990.48615	21.43
NYNFGGEFVEAMIR	28		0	85	7.06247E-08	2	1646.76042	-1.70	823.88385	31.03
DGVLEEQIER	2		0	84	1.2304E-07	2	1187.59026	-0.05	594.29877	16.33
SKATNDEIFSILK	4		1	83	9.39629E-08	2	1465.78948	-0.19	733.39838	19.52
mFDYTDDPEGPVmPGSHSVER	4	M1(Oxidation); M13(Oxidation)	0	83	9.65954E-08	2	2397.99077	-2.31	1199.49902	15.73
FLSDLVNcHVIAAPSMVAMFENFVSV	12	C8(Carbamidomethyl)	0	83	1.08882E-07	3	4078.97458	0.80	1360.32971	29.90
NHPQMIAVLVDKMIR	3		1	81	1.45531E-07	3	1764.96054	-0.39	588.99170	20.56

FLSDLVNcHVIAAPSmVAmFENFVSV	9	C8(Carbamidomethyl); M16(Oxidation); M19(Oxidation)	0	81	1.6142E-07	3	4110.96274	0.39	1370.99243	31.98
mFDYTDDPEGPVMPGSHSVER	9	M1(Oxidation)	0	80	1.79869E-07	2	2381.99614	-2.21	1191.50171	17.13
KDGVLEEQIER	11		1	80	2.97952E-07	2	1315.68498	-0.23	658.34613	14.25
ANNYNEAVYLVR	23		0	78	4.18412E-07	2	1425.71086	-0.90	713.35907	19.30
IEVFVQTLLHLAAK	105		0	77	4.43564E-07	2	1581.93645	0.06	791.47186	36.15
LQEKVESAQSEQK	5		1	76	6.58577E-07	2	1503.76457	-0.28	752.38593	9.91
cETDGTSVLTPWYK	4	C1(Carbamidomethyl)	0	74	7.6552E-07	2	1656.75725	-0.12	828.88226	20.53
ATNDEIFSILK	34		0	74	8.72355E-07	2	1250.67204	7.43	625.83966	26.97
LFVWEILHSTIR	40		0	72	1.19662E-06	2	1513.85234	-0.18	757.42981	23.72
TcAAQLVSYPGK	8	C2(Carbamidomethyl)	0	72	1.90788E-06	2	1294.64299	-2.36	647.82513	16.51
DVPNPNQDDDDDEGFSFNPLKIEVFV	4		1	70	1.89652E-06	4	3940.92519	0.27	985.98676	32.32
NYNFGGEFVEAmIR	3	M12(Oxidation)	0	69	2.36568E-06	2	1662.75688	-0.76	831.88208	20.43
SAcSLESNLEGLAGVLEADLPNYKSK	1	C3(Carbamidomethyl)	1	69	2.53488E-06	3	2765.36698	0.24	922.46051	26.18
NHPQMIAVLVDK	12		0	65	6.29443E-06	2	1364.73564	0.07	682.87146	16.93
FHEVFKTLAESDEGK	1		1	64	7.42945E-06	3	1736.84891	-0.09	579.62115	14.96
LQPGSLPQVLAQATEmLYMR	18	M16(Oxidation)	0	63	1.04221E-05	3	2262.16092	-0.56	754.72516	26.81
IFANTESYLK	25		0	63	1.15686E-05	2	1185.61638	1.12	593.31183	17.31
SFSHSFSALAK	7		0	61	1.72696E-05	2	1181.59575	0.64	591.30151	15.15
YGDESSNSLPGHSVALcLAVAFKSK	2	C17(Carbamidomethyl)	1	60	1.88346E-05	3	2637.30082	1.13	879.77179	20.24
NHPQmIAVLVDK	7	M5(Oxidation)	0	59	2.52433E-05	3	1380.72736	-2.25	460.91397	14.67
TcAAQLVSYPGKNK	2	C2(Carbamidomethyl)	1	55	7.7554E-05	2	1536.78459	0.42	768.89594	13.84
FImILTEHLVR	12	M3(Oxidation)	0	55	6.03888E-05	2	1387.76970	-5.03	694.38849	22.06
FVIEENLHcIIK	7	C9(Carbamidomethyl)	0	54	8.77035E-05	2	1514.80303	-0.40	757.90515	19.07
IQKELEEAK	2		1	54	0.000142391	2	1087.59953	0.10	544.30341	10.33
THVPmLQVWTADKPHPQEEYLDcLW	4	M5(Oxidation); C23(Carbamidomethyl)	0	52	0.000126461	4	3677.77578	-3.35	920.19940	22.26
SFSHSFSALAKFHEVFK	3		1	52	0.000135818	2	1968.99663	-0.05	985.00195	18.67
FLSDLVNcHVIAAPSmVAMFENFVSV	20	C8(Carbamidomethyl); M16(Oxidation)	0	51	0.000149265	4	4094.96495	-0.31	1024.49670	26.72
THVPMLQVWTADKPHPQEEYLDcLW	4	C23(Carbamidomethyl)	0	50	0.000182371	4	3661.79116	-0.55	916.20325	23.35
VESAQSEQK	1		0	48	0.000343492	2	1005.48454	-0.23	503.24591	6.34
FIMILTEHLVR	30		0	48	0.000310425	2	1371.78166	-0.08	686.39447	21.46
IEVFVQTLLHLAAKSFSHSFSALAK	2		1	44	0.000805298	4	2744.51259	-0.33	686.88361	26.99
HILRPYLAFDSILcEALQHNLPPFTPPP	9	C14(Carbamidomethyl)	0	44	0.000841311	5	4569.28022	-1.00	914.66187	25.15
RDWYVYAFLSSLPWVGK	7		1	43	0.001009152	3	2087.07749	1.20	696.36401	28.34
HILRPYLAFDSILcEALQHNLPPFTPPP	4	C14(Carbamidomethyl); M37(Oxidation)	0	40	0.001802837	6	4585.26135	-4.00	765.04962	24.70
TLAESDEGK	4		0	40	0.002382199	2	949.44695	-0.40	475.22711	10.74
KDAEMDR	1		1	40	0.001883461	2	864.38793	-0.14	432.69760	6.62
DAEmDRIFANTESYLK	1	M4(Oxidation)	1	40	0.001995063	3	1918.88761	1.25	640.30072	19.53
LQPGSLPQVLAQATEmLYmR	2	M16(Oxidation); M19(Oxidation)	0	40	0.002079489	2	2278.15874	0.71	1139.58301	26.34
VmFEVWR	6	M2(Oxidation)	0	40	0.002345397	2	982.48168	0.12	491.74448	18.81
ILRLLcTVAR	1	C6(Carbamidomethyl)	1	40	0.002147616	3	1214.73731	-2.41	405.58395	17.26
WSWEDWSDcLSQDPESPKPKFVR	1	C9(Carbamidomethyl)	1	39	0.002264418	4	2879.30214	-2.55	720.58099	21.70
IPLNYHIVEVIFAELFQLPAPPHIDVMY	7	C36(Carbamidomethyl)	0	39	0.002426367	4	4350.33510	0.66	1088.33923	33.82
NLFLVIFQR	20		0	38	0.003507168	2	1149.68035	2.09	575.34381	31.98
LFVWEILHSTIRK	3		1	36	0.004676884	2	1641.94597	-0.99	821.47662	21.60
RSDDDDR	1		1	35	0.006713617	2	878.35979	-0.14	439.68353	5.91
FVREVLEK	1		1	34	0.008648814	2	1019.59075	2.24	510.29901	12.91
VMFEVWR	13		0	34	0.011872954	2	966.48534	-1.35	483.74631	22.29
LSYHQR	8		0	33	0.012452194	2	803.41545	-0.52	402.21136	11.54
TSDANETEDHLESLIcKVGEK	1	C16(Carbamidomethyl)	1	33	0.010963685	3	2375.09433	-3.74	792.36963	19.17
LLcTVAR	5	C3(Carbamidomethyl)	0	33	0.01676474	2	832.46971	-1.52	416.73849	14.17
EVLEKcMR	1	C6(Carbamidomethyl)	1	32	0.016067012	2	1064.52324	0.44	532.76526	11.01
TQIVDcAAVANWIFSSELSRDFTR	1	C6(Carbamidomethyl)	1	31	0.014586684	3	2786.36002	1.17	929.45819	25.92
FVIEENLHcIIKSHWK	1	C9(Carbamidomethyl)	1	31	0.016179182	3	2053.06748	-0.67	685.02734	18.81
QLKESLK	1		1	31	0.020498012	2	845.51085	2.03	423.25906	9.70
NKIPLNYHIVEVIFAELFQLPAPPHIDV	3	C38(Carbamidomethyl)	1	30	0.018153341	4	4592.47182	0.37	1148.87341	31.89
ELEEAKEK	1		1	29	0.041133882	2	975.49663	-2.81	488.25195	10.52
DAEMDRIFANTESYLK	1		1	27	0.036054259	3	1902.88346	-3.59	634.96600	22.24
SHWKER	1		1	27	0.041586902	2	842.42644	-0.42	421.71686	7.04
SDDDDRSSDR	1		1	23	0.098618086	2	1167.45085	-0.06	584.22906	5.97
IPLNYHIVEVIFAELFQLPAPPHIDVmY	1	M27(Oxidation); C36(Carbamidomethyl)	0	22	0.131509331	4	4366.33315	1.37	1092.33875	32.27
FLSDLVNcHVIAAPSMVAMFENFVSV	1	C8(Carbamidomethyl)	1	21	0.153446352	4	4235.07046	-0.47	1059.52307	28.39
