# Supplementary material for: Long lasting control of viral rebound with a new drug ABX464 targeting Rev – mediated viral RNA biogenesis
Source: Retrovirology. 2015 Apr 9;12:30. doi: 10.1186/s12977-015-0159-3 (PMC4422473; doi:10.1186/s12977-015-0159-3)
Supplement: Additional file 7: Figure S5. — ABX464 pharmacokinetics parameters. a. Main plasma pharmacokinetic parameters of ABX464 and ABX464-N-glucuronide in the mouse after an administration of 40 mg/kg of ABX464 by the oral route. b. Plasma pharmacokinetic parameters of ABX464 and ABX464-N-glucuronide in the mouse after oral administration. Female OF1 mice received 40 mg/kg of an ABX464 suspension in Labrafil - DMSO 5% per os, once a day during 28 days. Blood was sampled at day 0, 7, 14, 21 and 28 (left panel) or 0, 0.5, 2, 4, 6, 9 and 24 hours after gavage at day 0 (right panel) (5 animals per time point). After day 28 the gavage was stopped in left panel (arrow shows end of the treatment). After 6 days of stop of the treatment (left panel) or 24 hours post treatment (right panel), neither ABX464 nor ABX464-N-glucuronide were still detectable. ABX464 and ABX464-N-glucuronide concentrations were quantified in plasma by LC-MS. c. Plasma pharmacokinetic parameters of ABX464 in in the marmoset monkey after one oral administration of 750 mg/kg of ABX464. [file 12977_2015_159_MOESM7_ESM.pdf]

a

	ABX464	ABX464-*N*-Glucuronide
C_{max} (ng/mL)	1164 ± 228	1957 ± 312
T_{max} (h)	1.0	1.3
$AUC_{(0-29h)}$ (ng.h/mL)	5454 ± 1423	11595 ± 2331

b

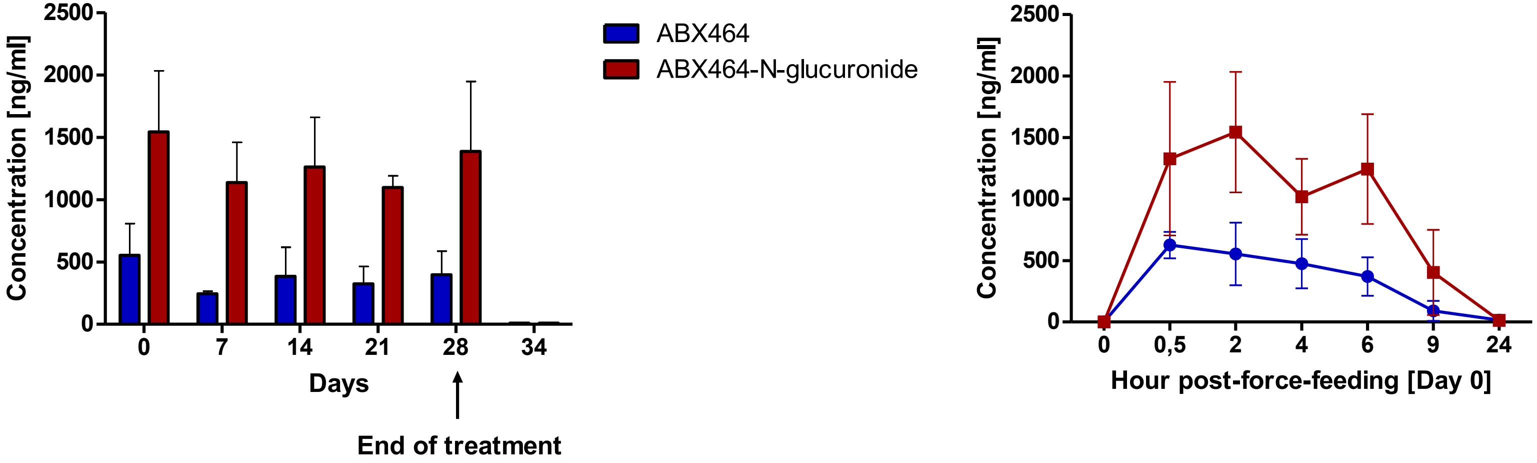

c

Parameter	ABX464		ABX464-*N*-glucuronide	
	Male	Female	Male	Female
C_{max} (ng/mL)	837 ± 258	465 ± 210	31450 ± 15203	26150 ± 11242
T_{max} (h)	3	2	4	3
$AUC_{(0-24h)}$ (ng.h/mL)	5478 ± 2080	4001 ± 399	262239 ±73106	153711 ± 45648
