# Supplementary material for: Long lasting control of viral rebound with a new drug ABX464 targeting Rev – mediated viral RNA biogenesis
Source: Retrovirology. 2015 Apr 9;12:30. doi: 10.1186/s12977-015-0159-3 (PMC4422473; doi:10.1186/s12977-015-0159-3)

a

Metabolites identified \ Species	Human	Mouse	Rat	Marmoset	Rhesus	Cynomolgus	Minipig	Dog
ABX464-*N*-glucuronide	1	1	-	1	1	1	-	-
Hydroxyl ABX464 glucuronide	-	1	-	1	1	1	1	-
Hydroxy-ABX464 metabolites	-	-	3*	2	2	1	-	3
Dihydroxyl-dihydro ABX464	-	1	-	-	-	-	-	-
N-Oxide	-	-	-	-	-	-	-	1
Unidentified minor metabolite	-	1	-	-	-	-	-	-

- : Not detected; * : 1 peak present in trace amounts.

b

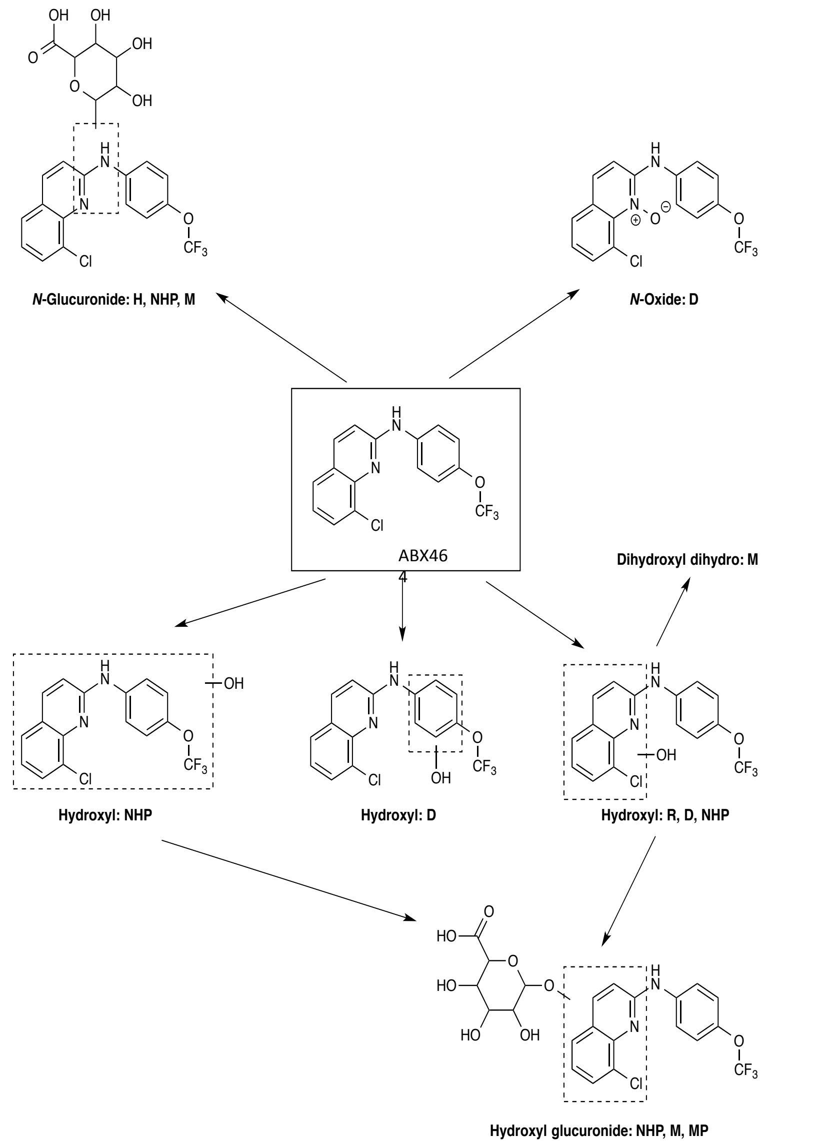

Supplement: Additional file 8: Figure S6. — ABX464 in vivo metabolism. a. In vitro metabolism profiling of ABX464 in cryopreserved hepatocytes primary cultures from various species. Hepatocytes were incubated with 10 μM ABX464 for 120 minutes at 37°C. Aliquots from each supernatant culture were analyzed by LC-MS/MS to identify potential ABX464 metabolites. The number of peaks present in the chromatogram is indicated for each metabolite in the corresponding species. b. Drawing and formula of ABX464 metabolites. [file 12977_2015_159_MOESM8_ESM.pdf]
