# Supplementary material for: Long lasting control of viral rebound with a new drug ABX464 targeting Rev – mediated viral RNA biogenesis
Source: Retrovirology. 2015 Apr 9;12:30. doi: 10.1186/s12977-015-0159-3 (PMC4422473; doi:10.1186/s12977-015-0159-3)
Supplement: Additional file 1: Figure S1. — Sequencing of HIV-1 genome after ABX464 treatment. a. HIV-1 strain YU2 was used to infect macrophages from 4 different donors (D1, D2, D3 and D4) and viruses from the supernatant of untreated (1–4) and treated (5–8) samples were harvested 8 days post-infection (pi) and subjected to deep sequencing using 454 pyrosequencing (according to GS junior method manual). Upper panel schematic representation of different ORFs encoded by HIV-1 genome. Position of all detected mutations/polymorphisms (POS). Corresponding codons altered by the mutations at each position (CODON REF/ALT) and expected amino acid changes (AA ref/Alt). Reference nucleotide (REF) the corresponding mutated nucleotide (ALT) at each position. In blue are the positions that were found to be mutated (the numbers indicate the frequency of each mutation). In red are the positions that are unaffected. b. Viruses from treated sample 5 in (a), was used to infect PBMCs from donor D3 and D4 and viruses from the supernatant of untreated (9 and 10) and treated (11, 12) samples were harvested 6 days pi and subjected to deep sequencing using 454 pyrosequencing (according to GS junior method manual). [file 12977_2015_159_MOESM1_ESM.pdf]

a

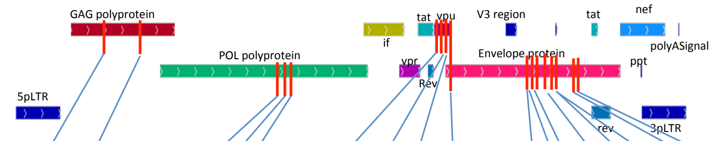

POS	1222	1852	3874	3876	3877	6066	6214	6215	6231	7553	7555	7556	7757	7758	7759	8008	8009
CODON REF/ALT	CAT/CCT	GGG/GTG	TAT/TAC	GTA/GGT		ATG(start)/CTG	GCA/GAG-GCG		Vpu: GAG/AAG<br>Env: ATG/ATA	ACA/ATA	AGA/CCA		TTG/TAG	TTG/TTA	GGA/AGA	ACT/TGT	
AA Ref/Alt	H/P	G/V	Y/Y	V/G		M/L	A/E-A		vpu: E/K<br>Env: M(start)/I	T/I	R/P		L/STOP	L/L	G/R	T/C	
REF	A	G	T	T	A	A	C	A	G	C	A	G	T	G	G	A	C
ALT	C	T	C	G	T	C	A	G	A	T	C	C	A	A	A	T	G
(1) D1 Ctl						99										84	85
(2) D2 Ctl						99				84	84	84		80	61		
(3) D3 Ctl						99											
(4) D4 Ctl	84	60	84	65	99	99	87	99	74								
(5) D1 treated with ABX-464						99							99				
(6) D2 treated with ABX-464						99											
(7) D3 treated with ABX-464						99											
(8) D4 treated with ABX-464						99		71									

b

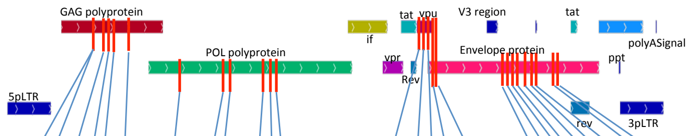

POS	1221	1222	1356	1357	1358	1852	2139	2963	2964	3874	3876	3877	6066	6214	6215	6231	6234	7536	7553	7555	7556	7719	7758	7759	8008	8009
CODON REF/ALT	CAT/AAT	CAT/CCT	AAC/CAC	AAC/AGG		GGG/GTG	NA	GAG/CTG		TAT/TAC	GTA/GGT		ATG(start)/CTG	GCA/GAG-GCG		Vpu: GAG/AAG<br>Env: ATG/ATA	Vpu: AGC/TGC<br>Env: AGA/AGT	ATT/ATA	ACA/ATA	AGA/CCA		AAA/AAG	TTG/TTA	GGA/AGA	ACT/TGT	
AA Ref/Alt	H/N	H/P	N/H	N/R		G/V	NA	E/L		Y/Y	V/G		M/L	A/E-A		vpu: E/K<br>Env: M(start)/I	vpu: S/C<br>Env: R/S	I/I	T/I	R/P		K/K	L/L	G/R	T/C	
REF	C	A	A	A	C	G	C	G	A	T	T	A	A	C	A	G	A	T	C	A	G	A	G	G	A	C
ALT	A	C	C	G	G	T	G	C	T	C	G	T	C	A	G	A	T	A	T	C	C	G	A	A	T	G
(9) Amp (5) on D3													81													
(10) Amp (5) on D4				84	75								99					76								
(11) Amp (5) on D3 treated with ABX-464								81	81				99													
(12) Amp (5) on D4 treated with ABX-464							87						99													
